# Research Progress in Ionic Liquid-Based Electrolytes for Electrochromic Devices

**DOI:** 10.3390/molecules30040973

**Published:** 2025-02-19

**Authors:** Hao Zhang, Yixuan Liu, Xuehan Wang, Keying Feng, Qilin Wang, Zheng Chen, Zhenhua Jiang

**Affiliations:** Key Laboratory of High-Performance Plastics, Ministry of Education, National and Local Joint Engineering Laboratory for Synthesis Technology of High-Performance Polymers, College of Chemistry, Jilin University, Changchun 130012, China; haozhang23@mails.jlu.edu.cn (H.Z.);

**Keywords:** ionic liquid-based electrolytes, polymeric ionic liquid-based electrolytes, electrochromic devices

## Abstract

Electrochromic (EC) technology has become one of the smart technologies with the most potential for development and application at this stage. Based on electrochromic devices (ECDs), this technology has shown extraordinary potential in the fields of smart windows, display devices, and sensing systems. With the optimization and iteration of various core components in ECDs, the electrolyte layer, a key component, evolved from its initial liquid state to a quasi-solid state and solid state. As driven by increasing application demands, the development trend indicates that all-solid-state, transparent electrolytes will likely become the future form of the electrolyte layer. Recently, the application of ionic liquid (IL)-based electrolytes in the field of electrochromism attracted a lot of attention due to their ability to bring outstanding EC cycling stability, thermal stability, and a wider operating voltage range to ECDs, and they are regarded as the new generation of electrolyte materials with the most potential for application. Although compared with conventional electrolytes, IL-based electrolytes have the characteristics of high price, high viscosity, and low conductivity, they are still considered the most promising electrolyte materials for applications. However, so far, there has been a lack of comprehensive analysis reports on “Research progress in ionic liquid-based electrolytes for electrochromic devices” within the EC field. In this article, the research progress of IL-based electrolytes in ECDs will be summarized from three perspectives: liquid, quasi-solid, and solid state. The future development directions of IL-based electrolytes for ECDs are discussed.

## 1. Introduction

Electrochromism refers to the phenomenon of reversible changes in transmittance, reflectance, and color of electrochromic materials under an applied voltage [1]. Electrochromism aroused the research interest of many researchers due to the fact that it only requires a low driving voltage to complete the modulation of color and transparency. Since the first appearance of electrochromic devices (ECDs) in 1969 [2], after years of development, ECDs have been used in smart windows, aircraft portholes, automotive anti-glare mirrors, smart displays, military camouflage, flexible devices, energy storage, and so on [3,4,5,6,7,8]. Electrochromic technology has become one of the smart technologies with the most development and application potential at this stage.

**Figure 1:**
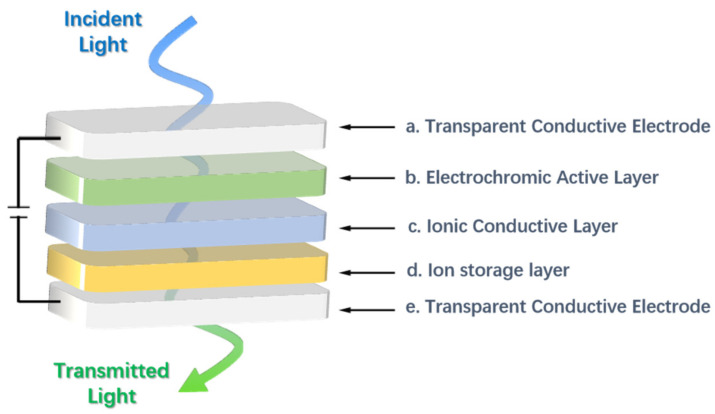
The structure of a typical electrochromic device.

High-performance ECDs are the basis for the rapid development and wide application of EC technology. In this paper, only the EC technology based on the electrochemical redox mechanism is discussed. The EC technology based on a polymer-dispersed liquid crystal mechanism has been commercially available, but it has not been widely studied due to the high driving voltage, complex production process, and other limitations [9]. At present, many types of ECDs have been developed, but they are derived from the classic sandwich structure. A typical electrochromic device is usually composed of the following five structures [10,11] (as shown in Figure 1, in order from bottom to top): (a) transparent conductive electrode (e.g., ITO-Glass, ITO-PET, etc.), (b) ion storage layer, (c) ionic conductive layer (electrolyte layer), (d) EC active layer, and (e) another transparent conductive electrode [12]. All of them are crucial for the construction of high-performance ECDs, Therefore, research on each layer is a central focus in this field. However, researchers generally agree that in ECDs, with the exception of the EC active layer, which determines the color change characteristics, the ion conduction layer has a wider choice and scope for development compared with other structural layers, both in terms of material selection and construction method. As in the case of EC active materials, there have been many reported research works on ionic conductive layers. The literature review on various electrolytes is also very rich and comprehensive.

In ECD, the ion transport layer, also called the electrolyte layer, which is usually located between the EC active layer and the ion storage layer, mainly plays the role of transporting ions and isolating electrodes. On the one hand, it allows the charged ions to move in a directional manner under the effect of an electric field to ensure that the EC active layer can achieve a controllable color (absorption spectrum) conversion through the doping detonation (redox) process of the ions. On the other hand, the electrolyte layer can also effectively isolate the EC active layer and the ion storage layer, preventing them from directly contacting and short-circuiting, ensuring the safe and stable operation of ECDs. It is found that the ion conduction layer has an important influence on the coloring efficiency, optical contrast, transmittance, response time, and cycle stability of ECDs [13].

During the development of electrochromic devices, three forms of electrolyte layers have been developed and applied one after another: liquid electrolyte, gel electrolyte, and solid electrolyte. To more intuitively illustrate the differences among these three types of electrolytes, we have provided examples of several reported electrolytes in Table 1. Liquid electrolytes: Liquid electrolytes used in ECDs are usually composed of solvents (e.g., water, propylene carbonate, vinyl carbonate, dimethyl carbonate, diethyl carbonate, etc.) and electrolyte salts (e.g., tetrabutylammonium perchlorate TBAP, as well as lithium salts, such as LiClO_4_, LiPF_6_, LiBF_4_, and LiCF_3_SO_3_, etc.), which have high ionic conductivity and a good wettability to electrodes. However, during the use of ECDs, the liquid electrolyte suffers from leakage and volatilization, which seriously affects the safety and service life of the ECDs, limiting their wide application in more fields.

Gel electrolyte: The gel electrolyte used in ECD is mainly a gel polymer electrolyte, which consists of a polymer matrix, electrolyte salts, and a solvent. On the one hand, the solvent plays the role of dissolving and dispersing the electrolyte salts; on the other hand, it plasticizes the polymer matrix. The solvent, together with the electrolyte salt, is filled into the internal spatial network of the polymer, which triggers the swelling phenomenon of the polymer, thus obtaining a semi-solid gel polymer electrolyte with a three-dimensional reticular structure. Both the polymer and the plasticizer are continuous phases. Although this electrolyte system is composed of liquid electrolyte components, the polymer network restricts the mobility and volatility of the solution electrolyte components; thus, the problem of leakage and volatilization of the liquid electrolyte is solved to a certain extent.

Solid-state electrolytes: Inorganic solid-state electrolytes [14,15,16,17,18,19], as well as polymer solid-state electrolytes [20,21,22,23,24,25,26,27], have begun to be investigated and employed in ECDs. Researchers are looking to replace conventional liquid electrolytes with such materials that have ionic conductivity in the solid state. Compared with traditional liquid electrolytes, in ECDs, solid-state electrolytes, due to the absence of liquid components, solved the solvent leakage problem of the device and the application safety problem at high temperatures on the one hand, and on the other hand, will also greatly simplify the assembly and production process of ECDs, which will contribute to the promotion of the development and application of electrochromic technology.

**Table 1:** Conductivity comparison of several liquid electrolytes, gel electrolytes, and solid-state electrolytes.

State	Content	Ionic Conductivity (S/cm)	Ref.
liquid	LiClO_4_/PC	/	[28]
	LiTFSI/PC	/	[28]
	[BMIM][BF_4_]	1.81 × 10^−3^	[29]
Gel state	PEGDA/PMMA/LiTFSI/PC	2.6 × 10^−4^	[30]
	PAM/KCl/H_2_O	6.4 × 10^−2^	[31]
	PVDF/LiClO_4_/PC	4.6 × 10^−4^	[32]
	PMMA/LiClO_4_/[Emim]BF_4_	2.9 × 10^−3^	[33]
Solid state	PMMA/KTFSI	5.7 × 10^−3^	[23]
	P(MEEMIm TFSI)	1.0 × 10^−5^	[34]
	LiPON	2.6 × 10^−6^	[35]
	Li_2.5_TaO_x_	1.0 × 10^−8^	[36]

Each of these three electrolytes mentioned above has its own advantages and disadvantages for use in ECDs. Although the specific values applied to ECD liquid electrolytes are not listed in the above table, there are literature reports that liquid electrolytes have the highest ion conductivity (10^−3^~10^−2^ S/cm) [37] and are the most extensively studied. However, due to the influence of solvent evaporation and leakage, the devices face great challenges in terms of safety and lifetime. Solid-state electrolytes offer higher safety and stability, and their use can avoid the leakage problems of liquid electrolytes. However, the research work on the use of solid-state electrolytes as ionic conductive layers is still in the exploratory stage due to their low ionic conductivity (10^−8^~10^−3^ S/cm) [13], which limits their wide use in practical applications. Gel electrolytes combine the advantages of liquid electrolytes and solid electrolytes, possessing high ionic conductivity (10^−4^~10^−2^ S/cm) [31] and good mechanical properties. However, most of the commonly used gel electrolytes currently use organic solvents, such as propylene carbonate, as the main component of the electrolyte. However, these solvents are highly volatile. Consequently, their evaporation at both ambient and elevated temperatures can cause irreversible damage to the electrolyte. This severely affects the thermal stability of the electrolyte and, moreover, the volatility of the organic solvents also impacts the long-term stability of the electrochromic devices. Therefore, it is very important to adopt electrolyte materials with better thermal stability and low volatility to improve the performance and reliability of ECDs.

**Figure 2:**
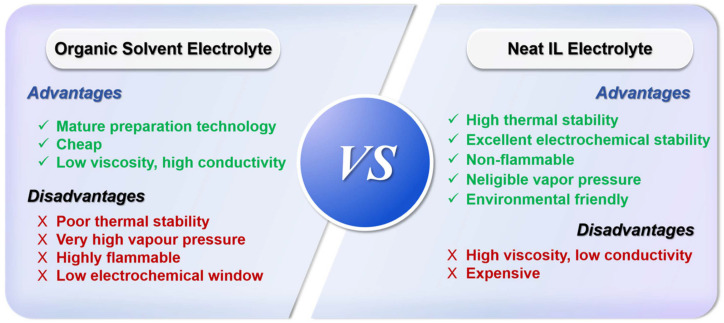
Comparison between organic solvent electrolytes and neat IL electrolytes.

In contrast, ionic liquid (IL) is an ideal substitute material for organic solvents in electrolytes [38] due to its high thermal stability (200~500 °C) [39,40], electrochemical stability (electrochemical window 4~6 V) [40], and negligible vapor pressure [41] (Figure 2). At present, the application of IL-based electrolytes in ECDs attracted high attention from researchers and a large amount of research work has been carried out. Current research results indicate that the use of ionic liquid electrolytes can improve the cycle life and environmental weather resistance of ECDs [42,43,44,45]. Moreover, IL can serve as not only an electrolyte in ECDs, but also as an electrochromic active material. Introducing units with color-changing activity into ILs through covalent bonds constructs electrochromic ILs. Thus, this approach endows ionic liquids with electrochromic properties. Therefore, IL-based electrolytes are one of the most promising types of electrolytes in the field of electrochromic technology.

However, up to now, there has been a lack of comprehensive introductions and analysis reports regarding the research progress of IL-based electrolytes in ECDs. This review aims to summarize the cutting-edge progress of ionic liquid electrolytes in the field of electrochromism, to sort out the advantages and characteristics of different types of IL-based electrolytes, and to discuss the challenges and future development trends in their applications in electrochromic devices. This will facilitate researchers in this field or those engaged in related research to gain a comprehensive understanding and quickly grasp the development status and future trends in this direction, thereby aiding further technological breakthroughs in this area.

## 2. Ionic Liquid Electrolyte

**Figure 3:**
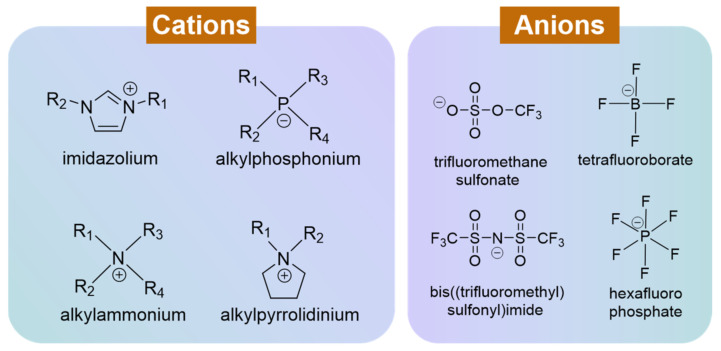
Common anions and cations in ionic liquids.

Ionic liquid (IL) [46] is a kind of ionic compound consisting entirely of anions and cations. Generally, ILs are salts formed by the combination of organic cations and organic anions (or inorganic anions). At relatively low temperatures (generally below 100 °C), IL is in a liquid state. There are four common cations: N,N′-dialkyl-substituted imidazolium ions, alkyl quaternary ions, alkyl quaternary ammonium ions, and N-alkyl-substituted pyrrolium ions. Common anions include trifluoromethanesulfonic acid ion, bis(trifluoromethanesulfonyl)imide ion, tetrafluoroborate ion, hexafluorophosphate ion, etc. (Figure 3) [47]. Although there are many types of ionic liquids, they should also be weighed and selected according to actual needs in the application process. For example, when selecting ionic liquids for use in ECDs, trade-offs should be made between IL viscosity, conductivity, transparency, cost, and compatibility with electrochromic materials.

Compared with conventional organic solvents, IL has excellent physical and chemical properties [48]: ionic liquids generally have low volatility, which allows them to remain stable at high temperatures or under vacuum conditions. Ionic liquids have a wide electrochemical window. Meanwhile, they can be used in a wide voltage range without electrochemical reaction, which gives them a wide range of applications in the field of electrochemistry. In addition, ionic liquids can be used to dissolve many organic and inorganic substances, which makes them an excellent reaction medium and solvent. The solubility of ionic liquids can be adjusted by choosing different combinations of cations and anions. Furthermore, most ionic liquids have high thermal stability and can be kept in a liquid state under high-temperature conditions, which makes them have application potential in the field of high temperature reaction and thermal catalysis.

Owing to their unique physicochemical properties, ionic liquids have been widely used in electrochemistry, chemical synthesis, separation technology, catalysts, drug delivery, environmental protection, wearable electronics, and so on [44,49,50,51,52,53,54,55,56]. Its development prospect is promising. Especially in the field of electrochemistry, as an electrolyte that is widely used in lithium-ion batteries [57,58,59], supercapacitors [60,61,62], fuel cells [63,64,65], and other energy storage and conversion equipment. In recent years, as people’s research on the application of IL-based electrolytes in the field of electrochromism deepened, its advantages in ECDs are also becoming more and more prominent. For example, ECDs that use IL-based electrolytes can achieve higher cycling stability, better optical contrast, higher thermal stability, and the ability to operate at low temperatures. All these features give IL-based electrolytes in electrochromic technology great prospects for development.

**Figure 4:**
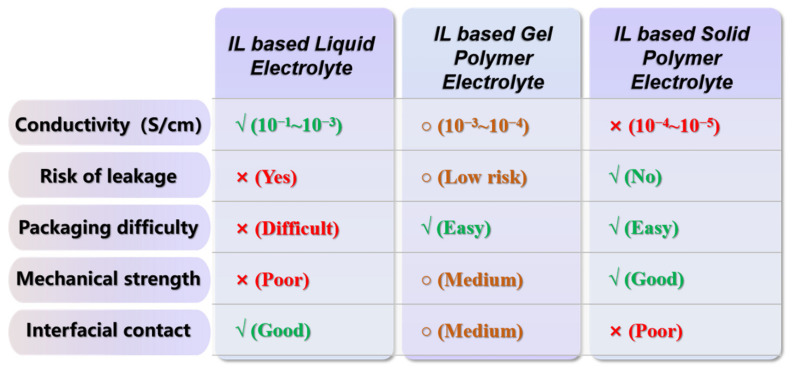
Characteristics of different types of IL-based electrolytes.

Similar to traditional electrolytes, IL-based electrolytes used in the field of electrochromism can be categorized into the following three types based on their composition and state: IL-based liquid electrolytes, IL-based gel polymer electrolytes (IL-GPE), and IL-based solid polymer electrolytes (IL-SPE). These three electrolytes also have their own advantages in practical applications (as shown in Figure 4). In this paper, the characteristics and applications of each type of electrolyte will be discussed in this order of classification.

### 2.1. IL-Based Liquid Electrolyte

Due to the large size and structural asymmetry of the anions and cations contained in ionic liquids, the anions and cations cannot be tightly stacked at the microscopic level, and the weak interactions between the anions and cations lead to the relatively low melting point of these materials, which are liquid at room temperature. In addition, this weak inter-ionic interaction also leads to the fact that the anions and cations in the ionic liquids can dissociate without the participation of other solvents, exhibiting excellent ionic conductivity (10^−5^~10^−2^ S/cm) [66]. Therefore, ionic liquids can be directly applied as electrolytes in the ion conduction layer of electrochromic devices without any solvent addition.

**Figure 5:**
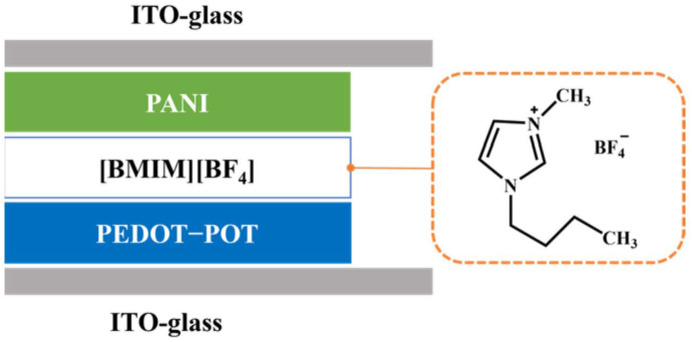
Electrochromic device (ECD) structure based on PANI-PEDOT-POT.

In the field of ECDs, IL-based electrolytes were first applied in the form of liquid electrolytes. In order to solve the problem of poor EC cycling stability of π-conjugated polymer EC smart windows (compared with inorganic ECDs), Mattes’ team carried out research work in 2002 from the perspective of an ionic conductive layer [42]. For the first time, IL-based electrolytes [BMIM][BF_4_] were used instead of aqueous electrolytes and conventional organic solvent electrolytes to construct an ECD with PEDOT-POT (poly(3,4-ethylene dioxythiophene)-poly(3-octylthiophene)) and polyaniline PANI as the EC active layers (transparent yellow to dark-blue interconverted, Figure 5). The ECDs exhibited excellent EC cycle stability (up to 10^6^ cycles) and extremely fast color change switching speed (1 ms). The EC cycle stability of the conductive polymer-based electrochromic device broke the highest record (3 × 10^5^ cycles) at that time. The research work in these ECDs was subsequently reported in more detail in a separate article [67]. The results of the team’s research show that the use of IL electrolytes as the ion conduction layer can play a crucial role in improving and enhancing EC cycling stability.

In 2017, Kim’s team constructed an ECD based on poly(3,4-ethylenedioxythiophene)/poly(styrenesulfonate) (PEDOT: PSS) using the ionic liquid electrolyte [EMIM][TFSI] [68]. With the same device structure (FTO glass/PEDOT: PSS/Electrolyte/ATO/FTO glass), the ECDs using [EMIM][TFSI] electrolyte exhibited a higher optical contrast (17–23%) compared with those using LiClO_4_ gel polymer electrolyte (optical contrast: 12%). The researchers believe that the key reason for the enhanced optical contrast of the ECDs was the higher conductivity of the [EMIM][TFSI] electrolyte (6.37 × 10^−4^ S/cm), which is approximately 3.5 times that of the LiClO_4_ polymer electrolyte (1.82 × 10^−4^ S/cm).

**Figure 6:**
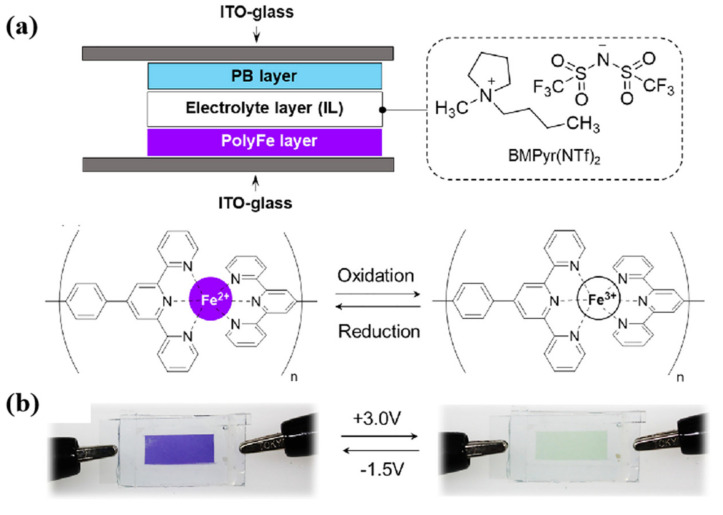
(**a**) Schematic presentation of the fabricated electrochromic (EC) device. (**b**) Color change in device redox by applying voltage +3.0/−1.5 V [43].

Due to the negligible vapor pressure and high thermal stability of IL electrolytes [38], they can address the issue of device failure caused by the volatility of traditional electrolytes in high-temperature environments, which is a common problem with conventional organic or aqueous electrolytes [29,43]. In 2019, Sanjoy Mondal’s team used the ionic liquid 1-butyl-1-methylpyrrolidinium bis(trifluoromethanesulfonyl)imide (BMPyr(NTf)_2_) as the electrolyte for ECDs and fabricated the ECD (Figure 6a) based on a Fe(II)-based metallosupramolecular polymer (poly Fe), with Prussian blue (PB) serving as the ion storage layer [43]. It was found that the ECD with IL electrolytes could not only achieve a color change from blue-violet to colorless at room temperature (at an operating voltage of +3.0/−1.5 V) (Figure 6b), but could also continue to complete 100 reversible EC cycles at room temperature after 80 cycles at a high temperature of 100 °C (chronoamperometry method). Although the optical contrast of the device reduced from 51% (first cycle) to 33% (last cycle), it still had a reversible EC change that could be seen with the naked eye. In contrast, LiClO_4_^−^ based gel electrolyte ECDs (after heat treatment at 100 °C) no longer have EC capability.

**Figure 7:**
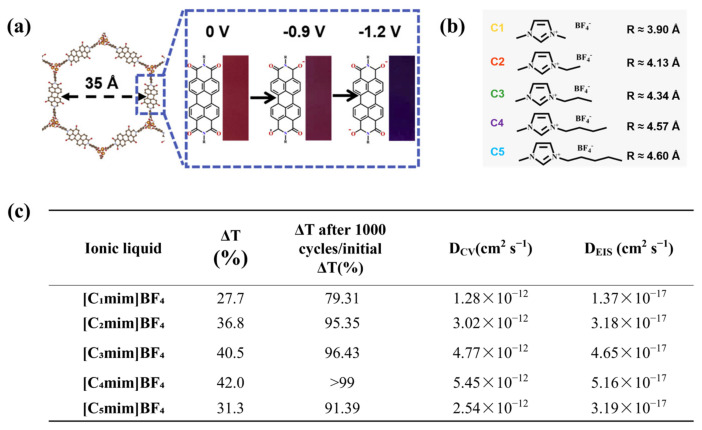
(**a**) Structure of Mg-PDI and its color changes under different voltages, transitioning from red to purplish-red and then to purple. (**b**) Chemical structural formula of ionic liquids of C1, C2, C3, C4, and C5. (**c**) The electrochromic properties and the ion diffusion coefficient of Mg-PDI thin films in different RTILs [29].

With the increasing number of studies employing IL as the electrolyte for ECDs, researchers found that selecting the best-matched ionic liquid electrolyte from the perspective of the ionic radius will result in better-performing ECDs. In 2022, Wang’s group developed a novel MOF electrochromic material, Mg-PDI (Figure 7a), with an intrinsic pore structure [29]. In order to obtain the ideal EC cycling stability, such ECDs used IL as the electrolyte. This study was also the first report of combining ILs with MOF for electrochromic applications. Unlike other works, the authors successively used four imidazolium-based ILs with similar structures but different sizes (C_1_[BF_4_], C_3_[BF_4_], C_3_[BF_4_], and C_4_[BF_4_], shown in Figure 7b), and determined that C_4_[BF_4_] (4.57 Å) was the most compatible ionic liquid electrolyte for Mg-PDI via a three-electrode test (1000 cycles, ΔT retention > 99%, Figure 7c). In addition, to investigate the reasons for the differences in EC characteristics between different IL electrolytes, the authors tested the diffusion coefficients of five ILs (Figure 7b) in Mg-PDI electrochromic films with COF structure using cyclic voltammetry (CV) and electrochemical impedance spectroscopy (EIS). After comparing the data shown in Figure 7c, they found that C_4_[BF_4_] exhibited the highest diffusion constant in Mg-PDI. Therefore, the authors believe that C_4_[BF_4_] is more suitable as an electrolyte for ECDs in this system. Based on these results, the authors further prepared the corresponding ECDs C_4_/Mg-PDI, which exhibited excellent EC cycling stability (500 cycles, ΔT retention rate > 95%), and the test results show that the C_4_/Mg-PDI devices could achieve reversible switching from red to purplish-red and purple in the temperature range of −40 °C to 150 °C, highlighting the excellent performance of IL electrolyte-based ECDs under extreme temperature conditions. The authors observed that the intrinsic pore structure (35 Å) of Mg-PDI provided channels for ion transport of ionic liquids and showed well-matched properties. As IL tends to have a large ionic radius, compared with the MOF materials with pore structure, it is difficult for IL to enter EC materials (e.g., inorganic materials and conjugated polymers) with small crystal spacing and small molecular gaps. Although by increasing the voltage, ions could enter the EC active layer and achieve electrochromism, many times embedding and disembedding of large cations will cause irreversible damage to the EC materials, which will reduce the cycling life of the ECDs. Therefore, the authors also prepared WO_3_ and P3HT-based ECDs (C_4_/WO_3_ and C_4_/P3HT), which were tested and characterized under the same test method. The test results show that after only 30 cycles, the electrochromic cycling stability of the C_4_/WO_3_ and C_4_/P3HT devices undergoes significant degradation, and reversible EC cycling cannot be achieved.

In addition to serving as electrolytes in electrochromic devices, researchers also hope that ionic liquids can be endowed with more functional properties, such as redox ionic liquids (RILs) electrolytes. Introducing units with redox activity into ionic liquids through covalent bonds will construct multifunctional redox-active ionic liquids [69,70], thereby endowing ionic liquids with electrochemical activity. These RILs, not only can play the role of ionic conduction in ECDs, but also have the function of electrochromism or balancing charge (reaction generated). The emergence of these RIL electrolytes demonstrates their huge potential for development and application in terms of simplifying the structure of ECDs and reducing production costs [71,72].

**Figure 8:**
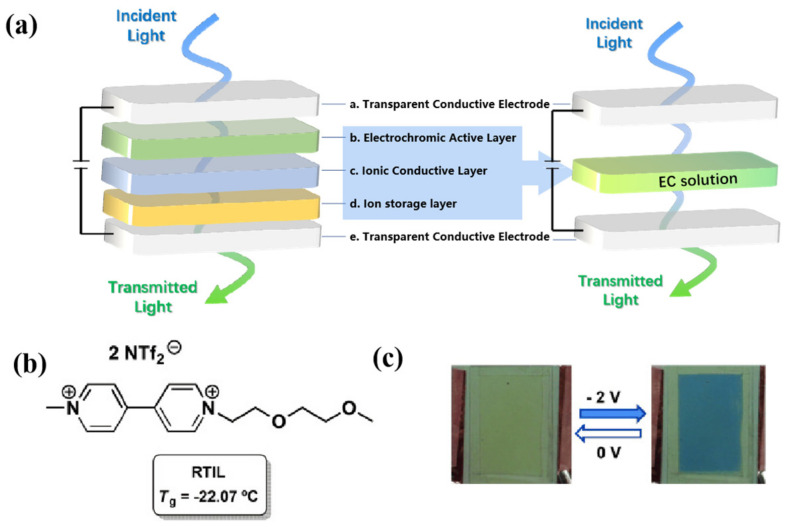
(**a**) Schematic diagram of the structure of five-layer and three-layer electrochromic devices. (**b**) the structure of [C_1_C_5_O_2_bpy][NTf_2_]_2_. (**c**) Reversible electrochromic device containing the electrochromic RTIL [C_1_C_5_O_2_bpy][NTf_2_]_2_ dissolved in the adequate electrolyte (50 mg mL^−1^) between two PET-ITO layers [75].

Since Branco et al. [73,74] reported the EC behavior of a series of ionic liquids with cobalt ethylenediaminetetraacetic acid complex as an anion and vanadium oxide as an anion in 2011, the possibility of IL acting as both an electrochromic material and electrolyte in ECDs was confirmed, which provides a new way to realize ECDs with simpler compositions. Specifically, ECDs could be achieved by simply encapsulating electrochromic ILs between two ITO glasses, without the need for additional electrolytes or EC materials, which greatly simplifies the structure and preparation process compared with the classic five-layer-structure ECD (Figure 8a). However, the above electrochromic ionic liquids have slow response times (t_c_ = 300~700 min, t_b_ = 270~483 min). Therefore, in 2014, Branco et al. prepared a series of non-symmetric disubstituted oxo-Bipyridinium ionic liquids by employing viologen, a more commonly used EC material in the field of electrochromism, as the redox unit of the ILs [75]. [C_1_C_5_O_2_bpy][NTf_2_]_2_ (Figure 8b) was used to assemble liquid ECDs due to its liquid state at room temperature and lower redox potential. With the authors reporting that this electrochromic IL-based ECD could achieve a reversible change from pale yellow to blue color in a short period of time (compared with their former work [73,74]), the voltages ranged from −2 to 0 V (cyclic voltammetry) (Figure 8c).

**Figure 9:**
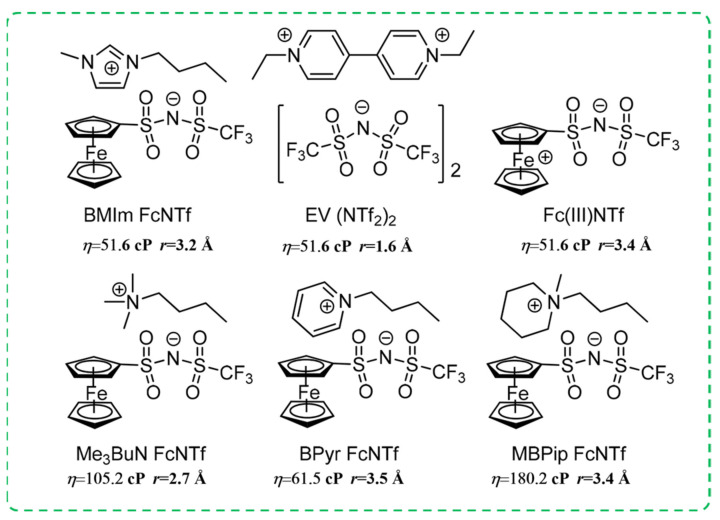
Chemical structure, viscosity, and solvated radius of redox ionic liquids based on ferrocenyl sulfonyl (trifluoromethyl sulfonyl) imide [FcNTf] with different counter cations: 1-butyl-3-methylimidazolium [BMIm], trimethyl butylammonium [Me_3_BuN], butyl pyridinium [BPyr], and 1-butyl-1-methylpiperidinium [MBPip]. Ferrocenium sulfonyl(trifluoromethyl sulfonyl)imide [Fc(III)NTf] and ethyl viologen di[bis(trifluoromethyl sulfonyl)imide] ([EV][(NTf_2_)_2_]) are also shown [78].

Previous studies have shown that the response time and driving voltage of viologen could be reduced by introducing ferrocene as a redox mediator in viologen-based ECDs [76,77]. However, ferrocene’s neutral nature makes it difficult to dissolve in ILs, thereby limiting its application as a redox mediator in ECDs with ILs as electrolytes. To enhance the solubility of ferrocene in ILs, Bruno’s team developed a series of new ferrocene-based redox ionic liquids in 2017 (Figure 9) [78]. According to the Stokes–Einstein equation, the diffusion coefficient is inversely proportional to viscosity and solvation radius. In contrast, BMIm FcNTf exhibited the highest diffusion rate (1.65 × 10^−7^ cm^2^/s) in ionic liquid [BMIm][NTf]_2_ due to its lower viscosity (51.6 cP) and smaller solvation radius (3.2 Å). Therefore, it was used for the assembly of the all-in-one ECDs (FcNTf/EV/IL-ECD). In FcNTf/EV/IL-ECD, [BMIm][NTf]_2_ serves as the redox mediator, ethyl viologen [EV][(NTf)_2_]_2_ as the electrochromic active material, and [BMIm][NTf]_2_ as the electrolyte. The FcNTf/EV/IL-ECD demonstrates short response time (t_c_ = 9.5 s, t_b_ = 5.5 s), good coloration efficiency (CE = 113.7 cm^2^/C), fairly optical contrast (ΔT = 38.8%), and excellent EC cycling stability (after 1000 cycles, ΔT retention rate is 94.8%).

**Figure 10:**
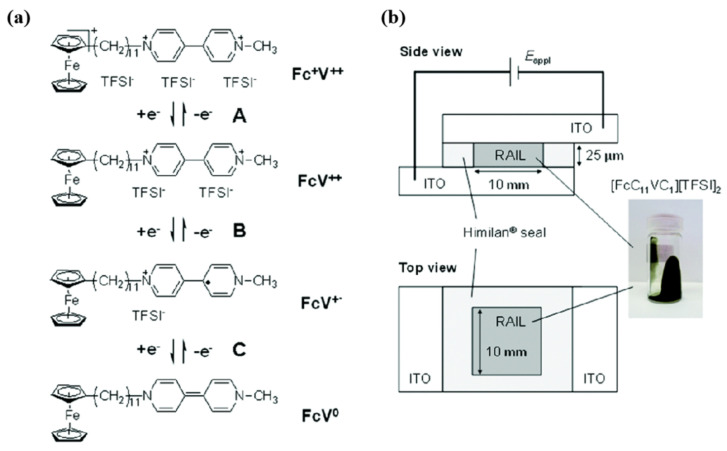
(**a**) Redox behavior of [FcC_11_VC_1_][TFSI]_2_. X^−^ stands for the TFSI anion. (**b**) Structure of the ITO two-electrode electrochromic cell [71].

In 2017, Hironobu’s team developed a new electrochromic ionic liquid [FcC_11_VC_1_][TFSI]_2_ (as shown in Figure 10a). Because the cationic part of this new ionic liquid contains both a viologen unit and a ferrocene unit, it could serve the triple functions of electrolyte, EC active material, and redox mediator. Therefore, [FcC_11_VC_1_][TFSI]_2_ was used to produce the ECDs without any additional components (such as other electrolyte salts or organic solvents, shown in Figure 10b) [71]. The ECD based on [FcC_11_VC_1_][TFSI]_2_ could change from light brown to dark purple at a voltage of 1 V and showed good coloration efficiency (91.4 cm^2^/C at 540 nm). Additionally, after 10,000 cycles (chronoamperometry), the current response of the device shows no significant change, demonstrating the excellent electrochromic stability of the ECD. The author argues that although [FcC_11_VC_1_][TFSI]_2_ greatly reduces the complexity of the liquid EC material, its high viscosity results in a slow coloring rate, and further optimization research on this kind of material is needed.

**Figure 11:**
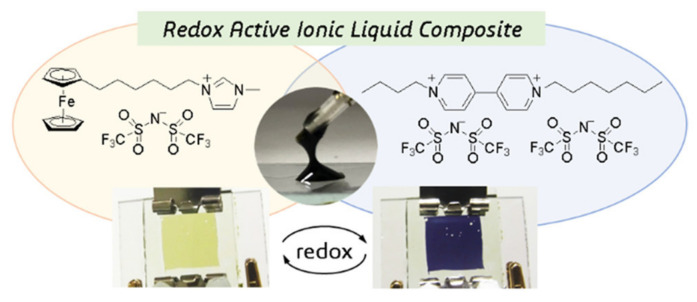
Chemical structure and physical photos of [C_4_VC_7_][TFSI]_2_ and [FcC_11_VC_1_][TFSI]_2_ [79].

Subsequently, in 2018, Hironobu’s team prepared a composite ionic liquid [79] composed of ionic liquids [C_4_VC_7_][TFSI]_2_ and [FcC_6_ImC_1_][TFSI] in an equal molar ratio (Figure 11). Compared with the previous work, the viscosity of the composite ionic liquid decreased significantly when the molar ratio of viologen to ferrocene was kept constant. The corresponding ECD test results show that under a 1 V voltage, the ECD could achieve a reversible color change, and the coloring efficiency is improved to 91.4 cm^2^/C.

**Figure 12:**
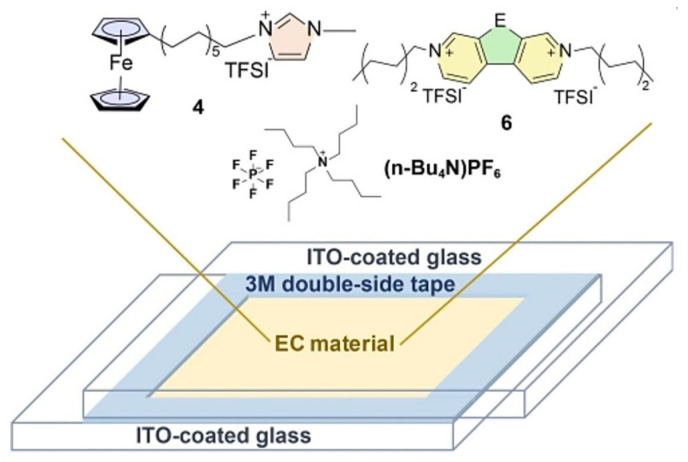
Schematic diagram of electrochromic devices based on [C_6_EVC_6_][TFSI]_2_, (E = S, Se, and Te) and [FcC_11_ImC_1_][TFSI] [80].

In 2021, Xiong’s team prepared a new EC ionic liquid composite material (as shown in Figure 12) using chalcogenoviologen-based ionic liquid ([C_6_EVC_6_][TFSI]_2_, E = S, Se, Te) and ferrocene ionic liquid ([FcC_11_ImC_1_][TFSI]) [80]. Several electrochromic composite ILs were dissolved in 0.05 M [nBu_4_N][PF_6_]/acetonitrile to prepare ECDs. The ECD based on [C_6_EVC_6_][TFSI]_2,_ (E = S) exhibited the best electrochromic properties. At −1.1 V and 0 V, the coloring and bleaching times were, respectively, 15.0 s and 23.4 s, with an optical contrast of 63.2%. After 3000 cycles, the ΔT retention rate of the device reached 98.2%, demonstrating excellent electrochromic stability. The authors consider that the introduction of chalcogenide elements could increase the rigidity and conjugation degree of the molecular structure. The larger atomic mass and higher polarization ability could promote the ion migration rate of compounds, thereby improving their electrochemical and electrochromic properties, significantly reducing the driving voltage of [C_6_EVC_6_][TFSI]_2_, and shortening the coloring time. It also shows that PET-based flexible ECDs made of [C_6_EVC_6_][TFSI]_2_ and [FcC_11_ImC_1_][TFSI] could be colored and discolored when applied with ±1.3 V voltage at the bending state.

**Figure 13:**
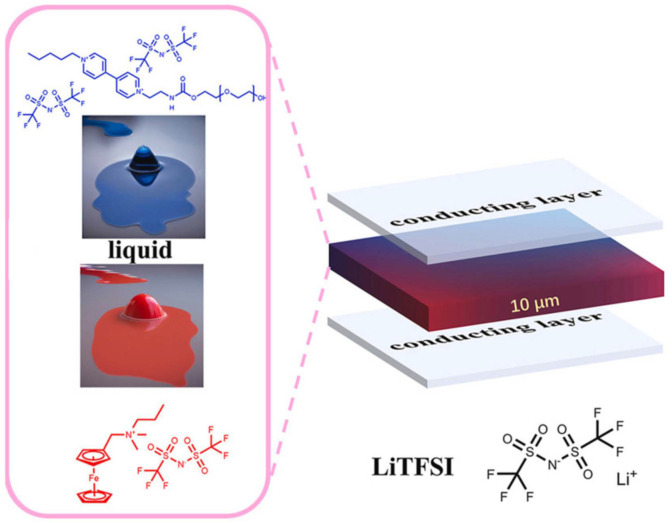
The structure of RTIL (Vio-IL and Fc-IL) and the ultra-thin (10 μm) ECD based on Vio-IL and Fc-IL [72].

Although many viologen-based ILs meet the strict definition of ionic liquids (melting point < 100 °C), a melting point higher than room temperature does not favor the application of viologen ILs in ECDs. In 2024, Xu et al. prepared two new room-temperature ionic liquids (RTILs) [72], viologen-based RTIL containing polyethylene glycol (vio-IL) and ferrocenyl RTIL (Fc-IL) (as shown in Figure 13). The introduction of long-chain polyethylene glycol makes vio-IL difficult to crystallize, and the large volume of [TFSI] in vio-IL helps to reduce the interaction between ions, thus making vio-IL liquid at room temperature. The ferrocenylmethyl dimethyl propylammonium bromide previously synthesized by the research group could also change from a solid state to a liquid state after [TFSI] ion exchange. The team prepared an ECD using [EMIm][NTf_2_] as the electrolyte, and vio-IL and Fc-IL as the electrochromic materials and redox mediator, respectively. The ECD exhibited a large optical contrast (ΔT = 79.4%) and excellent electrochromic cycle stability (1000 cycles, ΔT retention rate of 96.2%), with a coloring time of 23.6 s and a fading time of 9.5 s. Additionally, the team reported an ultra-thin (10 μm) ECD based on these two ionic liquids. The shorter ion transport path shortens the response time of the ECD. The ultra-thin ECD had a coloring time of 13.3 s and a fading time of 3.7 s, and it also showed good cycle stability (ΔT retention rate was 95.6% after 1000 cycles).

In addition to the application of ionic liquids based on viologen and ferrocene derivatives in ECDs, the TEMPO (2,2,6,6-tetramethylpiperidinyl-1-oxide) structure has also been introduced into ILs for applications in the field of electrochromism. TEMPO’s N-O• has the advantage of rapid electron transfer in electrochemical reactions due to its high intrinsic heterogeneous rate constant (about 10^−1^ cm/s), and nitroxyl free radicals (N-O•) are protected by their spatial structure, showing good chemical stability in organic solvents. Consequently, this structure has been incorporated into ionic liquids for applications in electrochromic devices.

**Figure 14:**
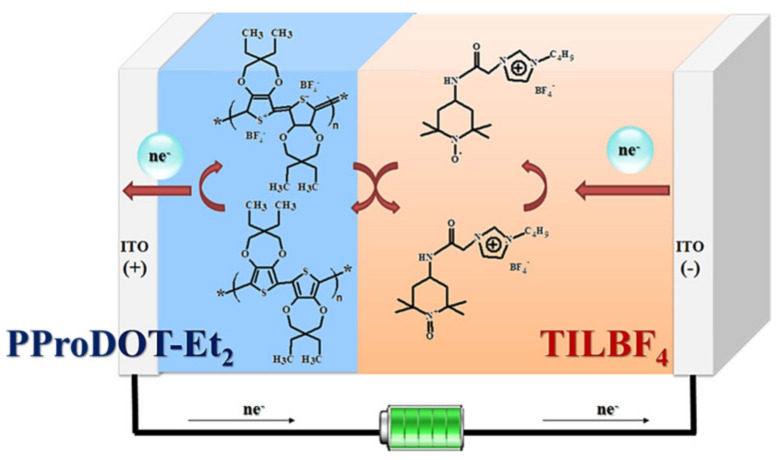
Schematic diagram of the proposed hybrid type ECD based on PProDOT-Et_2_ and TILBF_4_ [81].

Additionally, in 2017, Fan et al. [81] combined TEMPO with ionic liquids through chemical modification to prepare a novel ionic liquid 1-butyl-3-(2-oxo-2-((2,2,6,6-tetramethylpiperidin-1-oxyl-4-yl)amino)ethyl)-1H-imidazol-3-ium tetrafluoroborate (TILBF_4_). Poly (3,3-diethyl-3,4-dihydro-2H-thieno-[3,4-b] dioxepine) (PProDOT-Et_2_) were used as the EC materials. A PC solution of 0.01 M TILBF_4_ and 0.1 M LiClO_4_ was used as the electrolyte to prepare PProDOT-Et_2_/TILBF_4_ ECD (as shown in Figure 14). The prepared electrochromic device exhibited a high optical contrast (ΔT = 62.2%), good electrochromic cycling stability (ΔT retention ratio is 96.8%, after 1000 cycles), and a fast switching speed with a coloration time (t_c_) of 3.6 s and a bleaching time (t_b_) of 4 s (reaching 95% of the saturated ΔT), as well as superior coloring efficiency (983.0 cm^2^/C at 590 nm).

**Figure 15:**
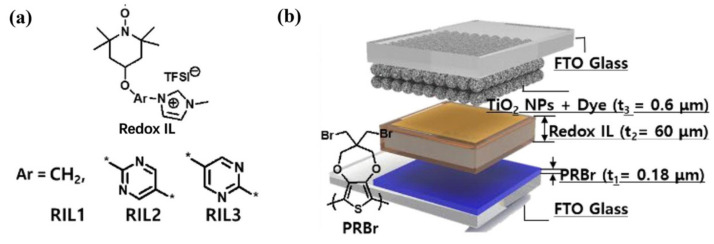
(**a**) The structure of RIL1, RIL2, and RIL3 (asterisks indicate the positions of the substituents). (**b**) Schematic diagram of the PECWs and chemical structure of the electrochromic polymer [82].

In 2022, Kim et.al. [82] synthesized three novel redox ionic liquids RILs (RIL1, RIL2, and RIL3) by connecting TEMPO units with imidazole cations through methylene or pyrimidine structures (as shown in Figure 15a). Due to their redox activity, these RILs could provide not only ion storage, but also redox mediators for the regeneration of dyes in the photoenergy harvesting process, and they could be ideal electrolytes for smart windows with energy storage and self-recharging functions in a single cell. The author used three types of RILs to manufacture three self-powered electrochromic capacitor windows (PECWs) (as shown in Figure 15b). Among them, the PECW based on RIL2 showed the best characteristics in terms of electrochromic performance and energy harvesting performance, with a photochromic efficiency of 65 cm^2^/W/min, an optical contrast of 90.8%, and excellent electrochromic cycle stability (basically no decay in ΔT after 4000 cycles).

The application of ionic liquids as electrolytes in ECDs can not only enhance the thermal stability, electrochromic cycling stability, and optical contrast of electrochromic devices, but also enables them to serve as both ion conduction layers and, through modification, electrochromic layers and ion storage layers. These studies clearly demonstrate the significant advantages of liquid IL-based electrolytes in electrochromic applications and also offer technical insights into the exploration of more structurally simplified ECD designs. For clarity, Table 2 presents a comprehensive summary of the key performance characteristics of IL liquid electrolytes and their corresponding ECDs. However, ECDs employing IL-based electrolytes as ion transport layers still encounter challenges related to device packaging and electrolyte leakage, which are attributed to their liquid nature at ambient temperature.

### 2.2. IL-Based Gel Polymer Electrolyte (IL-GPE)

Although IL-based liquid electrolytes have many application advantages in the field of ECDs, any liquid electrolyte with fluidity, whether in rigid or flexible devices, faces challenges related to device packaging and electrolyte leakage. At present, there are two approaches to solving this problem: one is to develop gel polymer electrolytes, and the other is to develop solid-state electrolytes. Compared with the latter, gel electrolytes, due to their higher ionic conductivity, have seen faster development and wider application. Gel polymer electrolytes can limit the fluidity of the liquid electrolyte, thus improving device safety [37]. Furthermore, gel polymer electrolytes offer lightweight and excellent viscoelastic properties, which provide significant advantages for the development of lightweight, thin, flexible, wearable, and large-area ECDs.

In IL-based gel polymer electrolytes (IL-GPEs), ILs exist in two forms: One is in a free state within the electrolyte, not covalently fixed in polymer networks, serving as a plasticizer and electrolyte salt; common polymer matrices, such as PEO, PMMA, PVDF, PU, PVC, etc., [33,83,84,85,86,87,88,89,90,91] are used to limit the mobility of the electrolyte. The other form involves the covalent attachment of ILs to a polymer backbone to form a polymeric ionic liquid (PIL), which serves as a polymer matrix to limit the mobility of the electrolyte and exhibits a specific level of ionic conductivity [92]. These two forms of ILs in IL-GPEs contribute differently to the overall performance of the electrolytes, influencing factors such as conductivity and mechanical stability. To provide clarity, in this section, we will analyze and introduce the development of polymer matrix materials in gel polymer electrolytes according to their types. In this section, we will analyze and discuss the development of IL-GPEs according to the types of polymer matrix materials. This classification helps to understand the unique contributions of each polymer matrix to the overall performance of gel polymer electrolytes. It can be divided into electrolytes based on polyvinylidene fluoride, poly (ethylene oxide), polyacrylate, PILs, and other polymer matrices.

#### 2.2.1. PIL-Based IL-GPEs

One way to develop ionic liquid gel polymer electrolytes (IL-GPEs) is to incorporate ionic liquids into polymers, forming PILs. These PILs not only retain their high ionic conductivity, chemical stability, and thermal stability, but also retain the processability of polymers, thereby overcoming challenges related to phase separation due to poor compatibility between ionic liquids and certain polymer matrices. These combined properties enable the PILs to effectively address issues of phase separation, making them suitable for a variety of ECDs. This type of PIL-based electrolyte also finds extensive application in electrochromic devices, as will be discussed below. In an effort to elucidate the key performance characteristics of PIL-based IL-GPEs and their associated ECDs, we have systematically collated the pertinent data, which are detailed in Table 3 (located at the end of Section 2.2.1).

**Figure 16:**
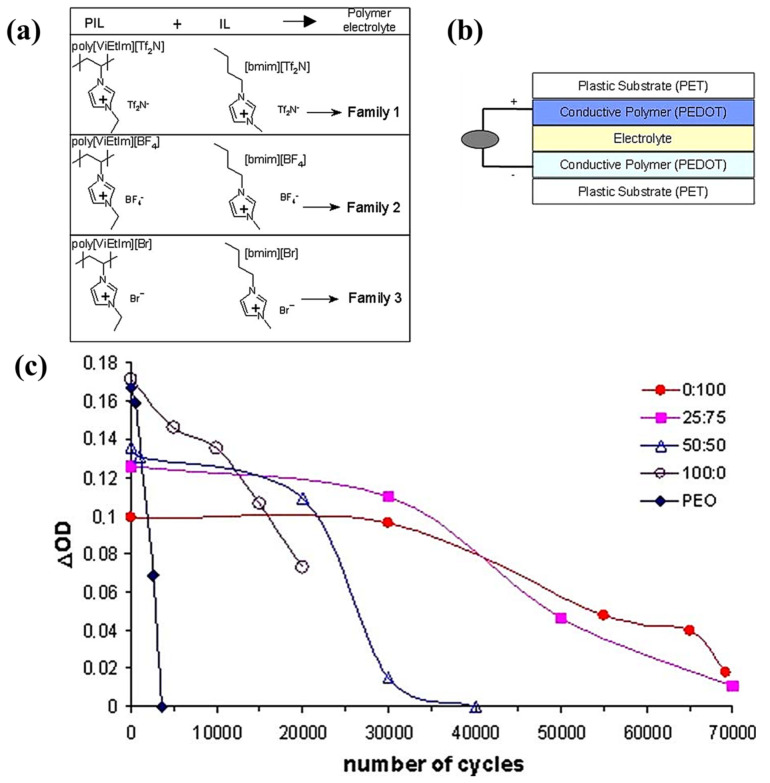
(**a**) Composition of the polymer electrolyte families. (**b**) Schematic diagram of electrochromic device structure based on PEDOT. (**c**) Variation in ΔOD as a function of the number of cycles for ECDs using polymer electrolytes with different ratios [emim][Br]:poly[ViEtIm][Br] and using the PEO/lithium triflate electrolyte [93].

In 2006, Marcilla synthesized a series of polyelectrolyte liquid electrolytes, including poly[ViEtIm][Tf_2_N], poly[ViEtIm][BF_4_], and poly[ViEtIm][Br] [93] (Figure 16a), through thermally initiated polymerization. These PIL electrolytes were synthesized. Subsequently, ionic liquids with analogous structures, such as [bmim][Tf_2_N], [bmim][BF_4_], and [bmim][Br], were used to prepare gel polymer electrolytes employed as the plasticizer. Considering the excellent electrochromic cycling stability exhibited by liquid ECDs based on [BMIM][Br] (symmetric devices with PEDOT as the EC layer), poly [ViEtIm][Br]-based ECD (symmetric devices with PEDOT as the EC layer) (Figure 16b) were selected from among these gel electrolytes for further assembly and performance evaluation. The test results indicate that, compared with the PEO/lithium salt-based gel electrolyte as the ion conduction layer (where ΔOD (optical density change) was reduced by 80% after 3000 cycles), the electrochromic cycling stability of this ECD was significantly enhanced, with ΔOD exhibiting a marked decrease after 3 × 10^4^ cycles (Figure 16c). This work represents the first application of a gel-based ionic liquid polymer electrolyte in the electrochromic field.

In 2008, Pozo Gonzalo et al. reported a PIL-based gel polymer electrolyte with a similar composition, focusing primarily on the application of symmetric PEDOT-based ECDs in near-infrared light modulation [94]. The ECD prepared using an electrolyte of poly (1-vinyl-3-ethylimidazolium tetrafluoroborate) and 1-butyl-3-ethylimidazolium tetrafluoroborate exhibited superior electrochromic performance, with a visible ΔT of 50.5% and an infrared ΔT of 44%, outperforming ECDs prepared using PEO-ClO_4_^−^ (which had a visible region ΔT of 39.9% and an infrared region ΔT of 3.3%). This ECD also demonstrated a coloring time of 2.7 s and a fading time of 3.8 s. As reported by the team, this work achieved the highest ΔT value in the infrared region for plastic-based ECDs utilizing conductive polymers at that time.

**Figure 17:**
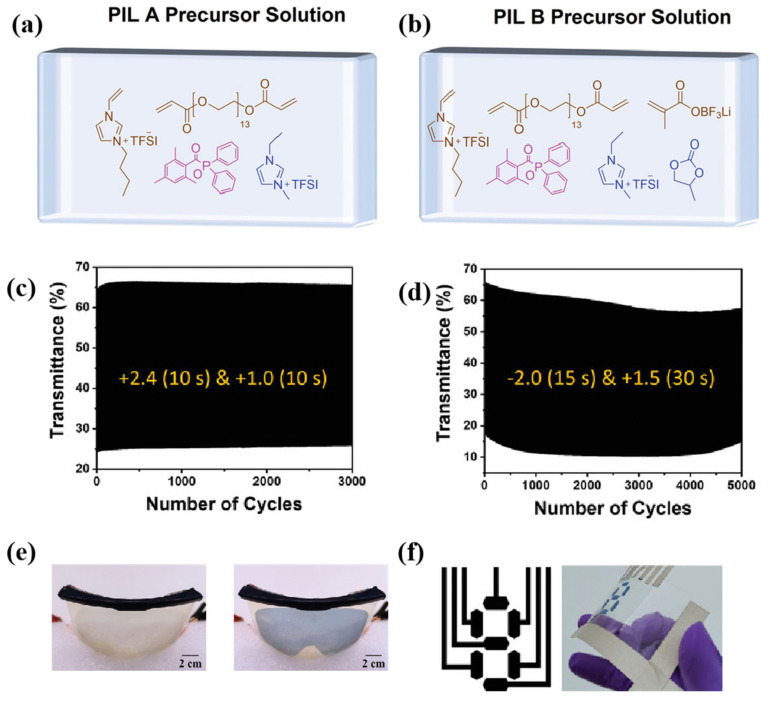
Composition of PIL A (**a**) and PIL B (**b**) precursor solutions (brown: stationary phase; blue: mobile phase; and pink: photoinitiator) (**c**). Changes in transmittance of the FeCP device at 572 nm recorded over 3000 cycles. (**d**). Changes in transmittance of the P-WO_3_ device at 660 nm recorded over 5000 cycles. (**e**) Top view a flexible electrochromic eye protector device captured in a photography lightbox to stimulate outdoor bright sunlight conditions. (**f**) Patterned digital arrays produced by wet etching approach to realize displays of various number patterns [95].

As the broad application of in situ polymerization technology in the field of gel electrolytes, researchers prepared gel polymer electrolytes based on a PIL matrix, achieving promising results. In 2022, Lee’s team prepared two gel electrolytes, PIL-A and PIL-B [95], based on PILs using an in situ photocuring process (Figure 17a,b). For PIL-A, the ethylene-functionalized ionic liquid VBIMTFSI and the acrylic end-capping crosslinking agent PEGDA700 undergo in situ polymerization under UV light irradiation at 395 nm, forming a three-dimensional PIL network structure. The ionic liquid EMIMTFSI (1,3-ethylmethylimidazolium bis(trifluoromethanesulfonyl)imide) serves as both an electrolyte salt and a plasticizer. PIL-B is derived from the formula of PIL-A, with the addition of PC as a plasticizer and vinyl-functionalized lithium salt (LiMA), incorporating the lithium salt structure into the three-dimensional network of the PIL through in situ polymerization. Respectively, PIL-A and PIL-B are suitable for ECDs with iron-centered coordination polymer (Fe-CP) and P-WO_3_ as the EC materials. Notably, the P-WO_3_-ECD using PIL-B as the ion conduction layer exhibited high electrochromic cycling stability, maintaining over 90% of its initial ΔT after 5000 cycles (Figure 17c,d). Based on these findings, the researchers developed a pair of curved eye protectors (Figure 17e) based on WO_3_ and a flexible digital display (Figure 17f), highlighting the significant advantages of PIL-based electrolytes in the fabrication of flexible ECDs.

**Figure 18:**
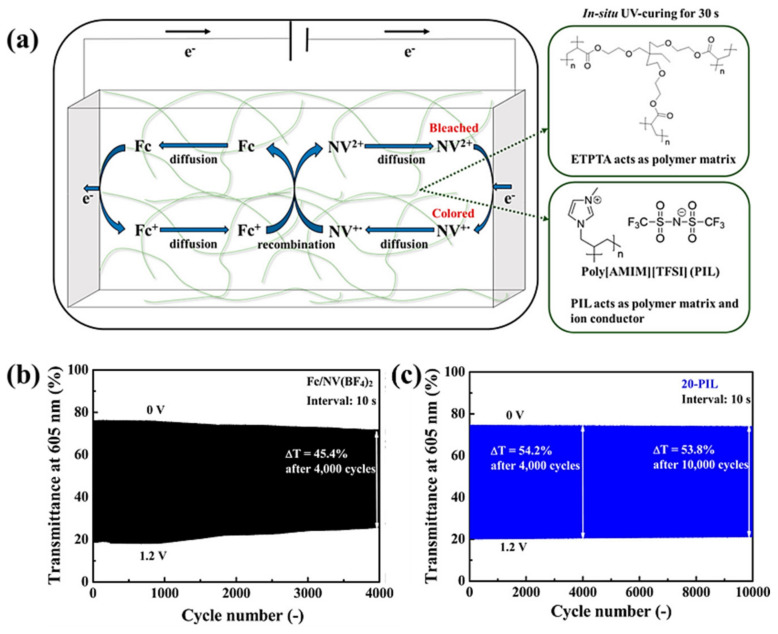
(**a**) Design concept and the working principle of the proposed PIL ECD. Long-term stability data for (**b**) the Fc/NV(BF_4_)_2_ ECD and (**c**) the 20-PIL ECD under continuous cycling between 1.2 and 0 V [44].

PIL-based IL-GPE can also be used for the preparation of integrated ECDs and demonstrates its unique advantages in integrated ECDs. In 2016, the Ho team developed a series of PIL-GPE featuring an interpenetrating network structure (Figure 18a) composed of polymer ETPTA and varying mass percentages of poly[AMIM][TFSI] (5-PIL, 20-PIL, 40-PIL, 60-PIL, 80-PIL) [44]. In addition, the researchers introduced the electrochromic material NV(BF_4_)_2_ and the redox mediator Fc into the gel electrolyte. This allows for a simple assembly of an “all in one” ECD by clamping the gel electrolyte between transparent electrodes. The test results show that the ECD based on the 20-PIL gel electrolyte exhibited the best electrochromic cycling stability, with a ΔT retention rate of 97.5% after 10,000 cycles, outperforming other PIL-GPE compositions (e.g., 80-PIL:4000 cycles, ΔT retention rate 77.7%) and liquid organic solvent electrolytes (4000 cycles, ΔT retention rate 80.1%) (Figure 18b,c). In addition, the researchers found that the electrochromic cycling stability of this type of ECD initially increased and then decreased with increasing polyelectrolyte content. They suggest that the higher cycling stability observed with 5-PIL and 20-PIL ECDs may be attributed to a reduced diffusion rate, which has been previously proved to positively affect the stability of viologen-based ECDs. However, as the PIL content increases further, despite the continued decrease in diffusion rate, the electrochromic cycling stability also significantly decreases. They speculate that one possible reason for this decline is the excessive polymer matrix capturing NV(BF_4_)_2_.

**Figure 19:**
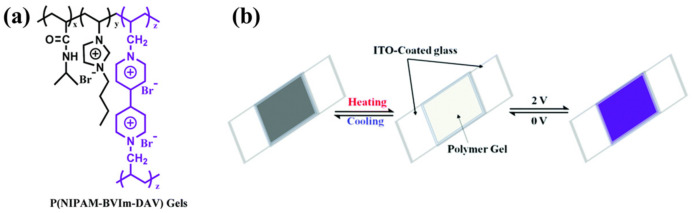
(**a**) Polymer structure of P(NIPAM–BVIm–DAV). (**b**) Schematic mechanism of thermochromic and electrochromic devices (TEDs) based on the prepared polymer gels [96].

In 2017, Chen et al. first reported a novel polyelectrolyte material P(NIPAMx-BVImy-DACVz) [96] that combines thermochromic and electrochromic functions (as shown in Figure 19a). It is a cross-linked polymer prepared by ternary copolymerization of N-isopropylacrylamide (NIPAM), 3-butyl-1-vinyl-imidazolium bromide [BVIm][Br], and diallyl viologen (DAV). In this polymer, the P(NIPAM)x segment exhibits thermochromic properties, the P(BVIm)y segment has ion conductivity, and the P(DACV)z segment has electrochromic properties and is responsible for constructing cross-linking structures between polymer chains. The IL-GPE, prepared with this PIL and water as a plasticizer, was sandwiched between ITO glass to fabricate thermochromic electrochromic devices (TEDs). These TEDs could achieve over 20 reversible transparency switching cycles between 20 °C and 33 °C and also exhibit reversible electrochromic (EC) changes from colorless and transparent to blue-purple under an applied voltage of 2 V (Figure 19b). The coloring and fading times are approximately 3 s and 5 s, respectively, and after 100 cycles, the current response shows minimal variation, suggesting high electrochemical cycling stability.

**Figure 20:**
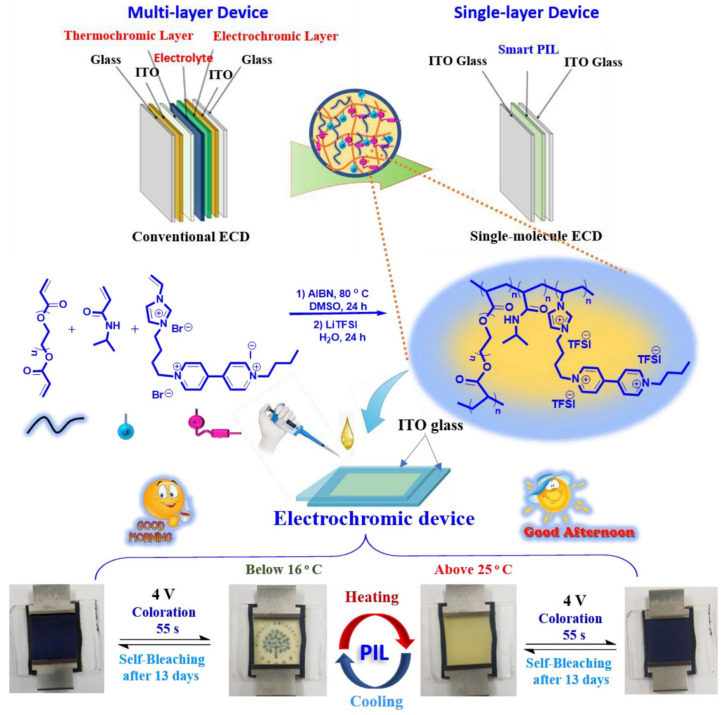
Schematic of the integrated thermochromic and electrochromic device using one single-molecule Smart PIL [97].

In 2021, Kim’s team also conducted similar research and prepared a new type of PIL material, Smart PIL [97], that combines thermochromic and electrochromic functions. In the structure of this cross-linked polymer, PEGDA segments are responsible for ion transport and constructing cross-linked networks; the P(NIPAM) segment exhibits thermochromic properties; and polymer segments containing viologen units display electrochromic properties (Figure 20). With water as a plasticizer, Smart PIL was formulated as an IL-GPE, which was then sandwiched between ITOs to fabricate a thermoelectric double-responsive smart window (Figure 20). TEDs could complete more than 20 reversible transparent switches between 16 °C and 25 °C. In terms of electrochromic performance, TEDs could also realize the reversible cycle from colorless transparent to blue under the application of voltage (4 V). These reversible switches and color changes demonstrated the device’s robust electrochromic capabilities. The optical contrast change in the prepared electrochromic device is 66.92%. Although the coloring time of 55 s is not ideal, it is worth noting that the device had an ultra-long memory effect. Under the voltage-off state, it takes the device 13 days to completely bleach.

**Figure 21:**
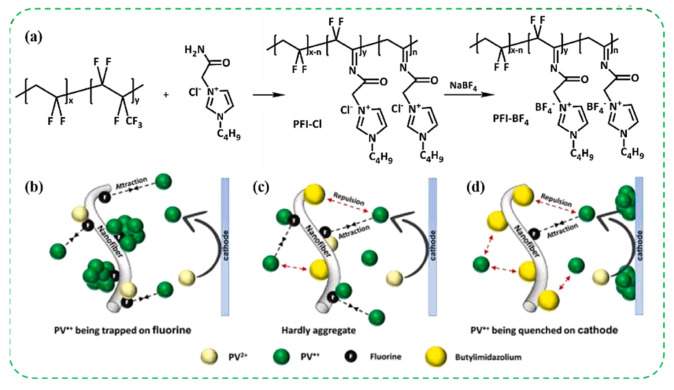
(**a**) The synthesized processes of PFI-BF_4_. (**b**–**d**) The sketches of the molecular interaction between the viologen ions and the PFI-BF_4_^−^ based membranes. (**b**) The raw PVDF-HFP membrane possessed a large number of lone-pair electrons of fluorine atoms, which provided strong attractive interaction for the PV^•+^ ions being trapped and aggregated on the membrane. (**c**) The PFI-BF_4__1.5 membrane exhibited the optimal molar ratio of imidazoliums to fluorines; the former supplied repulsive interaction, and the latter offered attractive interaction towards the PV^•+^ ions. The equilibrium electrostatic interactions established among imidazoliums, fluorines, and PV^•+^ ions could render the ECD with fewer viologen aggregation. (**d**) The PFI-BF_4__2 membrane had excess imidazoliums, which provided overwhelming repulsive interaction that caused the PV^•+^ to be quenched on the cathode [45].

In 2022, the He team designed and synthesized a novel polymer called poly (vinylidene fluo-ride-co-difluoroethyleneaminooxyethyl-1-butylimidazolium-co-vinylaminooxyethyl-1-butylimidazolium tetrafluoroborate) (PFI-BF_4_) (Figure 21a). After electrospinning PFI-BF_4_ into a film, it was immersed into a PC solution containing 0.05 M phenyl viologen (PV) and 0.05 M Fc to obtain gel electrolyte [45]. This kind of gel was sandwiched between ITO glass to form an ECD. Compared with the ECD using PVDF-HFP (ΔT = 68.9%, t_b_/t_c_ = 5.6/1.6 s) as the polymer matrix, the ECD based on PFI-BF_4_ showed more excellent optical contrast and switching time (ΔT = 72–74.6%, t_b_/t_c_ = 3.5–4.3/2.6–3.3 s). This is because the PFI-BF_4_^−^ based electrolyte had more excellent conductivity (8.61–9.28 × 10^−3^ S/cm) than the PVDF-HFP-based electrolyte (5.94 × 10^−3^ S/cm). The ECD based on PFI-BF^−^ 1.5 showed excellent electrochromic stability (maintaining 95.9% of its initial ΔT value after 10,000 cycles and 88.3% of its initial ΔT value after 50,000 cycles), while the ECD based on PVDF-HFP exhibited rapid decay within 10,000 cycles. They believe that this is due to the balanced repulsive and attractive interactions provided by PFI-BF_4_^−^ 1.5 for the migration of PV⁺ ions so that PV⁺ ions will not be captured by fluorine nor undergo dimerization and quenching (Figure 21b–d).

**Figure 22:**
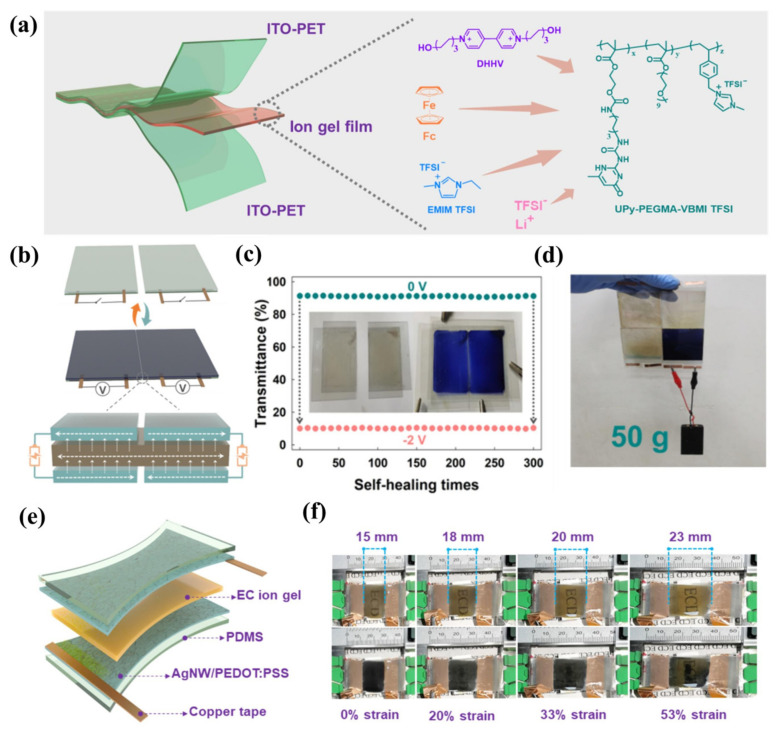
(**a**) Chemical structures of the constituents of electrochromic ion gels and a demonstration diagram of the single-layered flexible ECDs containing the ion gel. (**b**) Schematic diagrams illustrate the self-healing and coloring processes of modular flexible ECDs. (**c**) Transmittance changes in neutral and colored states of modular flexible ECDs after 0 to 300 cycles of separation and self-healing processes. (**d**) A photo image featuring four modular flexible ECDs (6 × 5 cm^2^) supporting a weight of 50 g after re-joining and coloring. (**e**) The demonstration diagram of the stretchable electrochromic devices containing the ion gel. (**f**) Photos of the stretchable ECDs (2 × 1.5 cm^2^, 0 and −2.5 V) at 0%, 20%, 33%, and 53% strain states [98].

To meet the application requirements of ECD in the field of flexible wearables, in addition to improving the flexibility of the device, it is also desirable that the core components of the device have a self-healing ability under certain conditions, especially the polymer electrolyte layer. In 2024, Kim’s team designed and developed a stretchable, self-adhesive, and self-healing PIL material (UPy-PEGM-VBMI-TFSI) [98]. The self-complementary quadruple hydrogen bonds (QHB) formed by UPyMA endow the polyelectrolyte electrolyte with self-healing properties and excellent mechanical properties, while VBMI endows the polymer network with ion conductivity. The mobile ions of PEGMA are transported through the soft phase of the PEGMA chain. Subsequently, based on the aforementioned PIL material, the research group used PC and EMIMTFSI as plasticizers, DHHV as EC material, Fc as redox mediator, LiTFSI as an electrolyte salt, and A PIL-based IL-GPE membrane (the ion gel film shown in Figure 22a) integrating EC and ion transport was prepared. The gel electrolyte was sandwiched between two layers of ITO-PET, and the all-in-one flexible ECD showed excellent EC performance. In addition, the flexible ECD also exhibits self-healing properties. Through self-healing and self-adhesive functions, interconnected modular large-area ECDs (38 × 40 cm^2^) have been achieved, providing insights into constructing devices with expandable areas (Figure 22b). More importantly, the PIL-based IL-GPE membrane could also be applied to the stretchable ECD (Figure 22c). Even under 53% strain, the ECD could still show 42.7% optical contrast, fast switching time (8.4 s for coloring, 13.5 s for fading), and ΔT = 41.6% after 600 cycles, exhibiting excellent electrochromic stability. After 100 cycles of tensile/recovery testing (between 0 and 40% strain), the device still maintains excellent electrochromic cycling stability, with a ΔT of 30%.

**Table 3:** The performance of PIL-based IL-GPEs and ECDs.

Electrolyte Membrane			Performance of ECD						
Electrolyte	Conductivity (S/cm)	Thickness (μm)	ECM	Color Changes	t_c_/t_b_ (s)	ΔT (%)	CE (cm^2^/C)	Cycles	Ref.
poly[ViEtIm][Br] /[BMIM][Br]	10^−5^~10^−2^	/	PEDOT	/	/	/	/	7 × 10^5^	[93]
poly[ViEtIm][BF_4_] /[BMIM][BF_4_]	/	/	PEDOT	/	3.6/2.7	44(IR)	/	/	[49]
PILA	/	1000	FECP	transparent to purplish-blue	1.5/1.9	45.2	/	3000	[95]
PILB	/	1000	P-WO_3_	colorless to dark blue	6.4/1.7	56.4	/	3000	[95]
Poly[AMIM][TFSI]	/	/	NV(BF_4_)_2_	colorless to dark blue	2.1/2.13	55.2	273.5	10^4^	[44]
P(NIPAMx-BVImy-DACVz)	/	/	P(NIPAMx-BVImy-DACVz	colorless to transparent purple	5/3	/	/	100	[96]
PIL-6	/	60	PIL-6	transparent to blue	t_c_ 55 s	/	73.1	/	[97]
PFI-BF_4__1.5	/	/	PFI-BF_4__1.5	transparent to green	3.9/1.7	73	460	5 × 10^4^	[45]
UPy-PEGM-VBMI TFSI	3.84 × 10^−3^	/	DHHV	colorless to dark blue	5.1/5.3	42.7	443.83	300	[98]

#### 2.2.2. Polyoxyethylene-Based IL-GPEs

Polyethylene oxide polymer is one of the most important polymer matrices in polymer electrolytes. It mainly includes two types of polymers: polyethylene oxide (PEO) and polyethylene glycol (PEG). Polymers with a molecular weight below 20,000 g/mol are referred to as PEG, while those with a molecular weight above 20,000 g/mol are referred to as PEO [99]. Polyethylene oxide polymers are widely used in the preparation of electrolytes for electrochemical devices due to their simple synthesis, low cost, and good processability [100]. These advantages make polyethylene oxide polymers ideal candidates for various electrochemical applications. At the same time, the soft backbone of these polymers endows them with good flexibility, making them promising in providing mechanical stability for flexible electrochromic devices. Some researchers combined IL with polyoxyethylene to carry out related work on ionic conductive layers [83,101,102,103,104,105,106,107,108]. In the next section, we will introduce polyoxyethylene gel polymer electrolytes containing ionic liquids. In an effort to elucidate the key performance characteristics of polyoxyethylene-based IL-GPEs and their associated ECDs, we have systematically collated the pertinent data, which are detailed in Table 4 (located at the conclusion of Section 2.2.2).

1. PEO-based IL-GPEs.

In 2007, Brazier’s team developed a PEO-based gel polymer electrolyte, P(EO)_10_LiTFSI + 0.96 PYR_14_TFSI [101]. Unlike conventional PEO gel electrolytes, it used the ionic liquid PYR14TFSI as a substitute for propylene carbonate (PC). This innovative substitution enhances the performance of the electrolyte, as evidenced by the following ECD application. Based on the P(EO)10LiTFSI + 0.96 PYR_14_TFSI, the team developed an ECD using WO_3_ as the electrochromic material and V_2_O_5_ as the counter electrode. This ECD had an optical contrast of 40–50% and still exhibits electrochromic ability after 1000 cycles, achieving an optical contrast of 28%. In addition, it is worth noting that this work represents the first application of electrolytes combining lithium salts with ionic liquids in the field of electrochromism. This pioneering work provides important insights and assistance for the preparation and development of ionic liquid-based electrolytes used in the field of ECDs.

In 2010, Desai’s team also reported an ionic liquid gel electrolyte (PEO)10LiTFSI: 25% P14TFSI [103] with a similar composition. It was used to assemble complementary electrochromic devices (PEDOT/PPY ECD) based on PEDOT/PPY. The test results show that the PEDOT/PPY ECDs prepared with (PEO)10LiTFSI: 25% P14TFSI (ΔT = 43%) exhibit lower optical contrast than the gel electrolyte (ΔT = 45%) using EC/PC as plasticizers. They believe that this is due to the higher ionic conductivity (1.00 × 10^−4^ S/cm) of 75% (PEO)8LiTFSI: 25% PC/EC (2.74 × 10^−4^ S/cm) compared with (PEO)10LiTFSI: 25% P14TFSI. However, the ECD prepared using IL-based electrolytes had excellent cycling stability (after 10^4^ cycles ΔT is still 43%). In contrast, PEDOT/PPY ECD based on 75% (PEO)8LiTFSI: 25% PC/EC showed a significant decrease after 10,000 cycles, with ΔT = 37%. This demonstrated the unique advantage of IL as an electrolyte in ECD.

**Figure 23:**
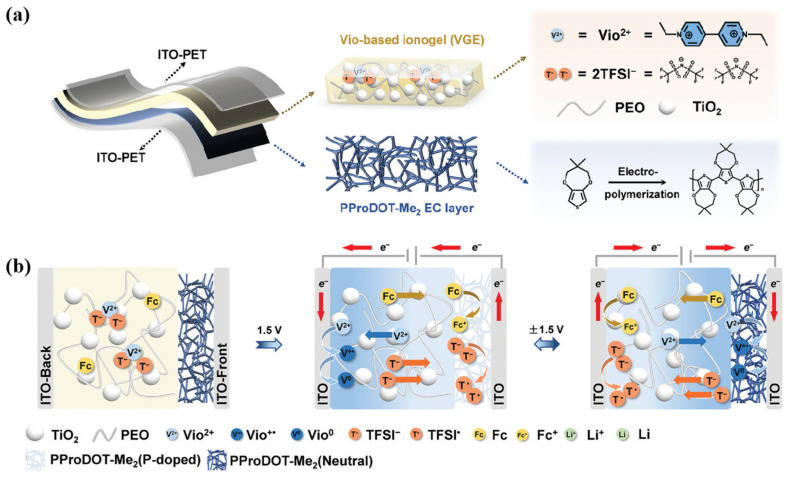
(**a**) Device structure of the VGE-based ECD. (**b**) Schematic diagrams of EC mechanism of the V-ECD at the initial, bleached, and colored states [83].

In 2024, Bai et al. reported an ionic gel (VGE) [83] based on divalent viologen cation. In VGE, PEO serves as the polymer matrix, PC as the plasticizer, TiO_2_ provides a nearly white background for ECDs, EtV(TFSI)_2_ serves as the electrolyte salt, and Fc serves as the redox mediator (Figure 23a). Additionally, a V-ECD based on PProDOT-Me_2_ as an electrochromic material was prepared using VGE. Compared with LGE using monovalent Li⁺ as the transport ion (ΔR = 13.3%), V-ECD prepared based on VGE exhibits higher optical contrast (ΔR = 39.4%). They believe that this is due to the presence of divalent viologen cations (Vio_2_⁺), which facilitates the insertion and extraction of a greater number of TFSI anions in PProDOT-Me_2_ compared with Li⁺. In addition, as Vio_2_⁺ is an electrochromic cation, while PProDOT-Me_2_ is bleached, Vio_2_⁺ could be reduced on the opposite side of the ECD (opposite side of the PProDOT-Me_2_ layer), thereby achieving color change on the opposite side of the ECD (as shown in Figure 23b). In their follow-up, the team prepared a series of EC gels capable of red, blue, and green changes by introducing viologen derivatives capable of different color changes into similar systems [108]. Based on this EC gel, all-in-one ECDs were assembled, which showed good electrochromic performance. These EC gels, with their versatile color-changing capabilities, enable the fabrication of simple displays and laid a foundation for the development of electrochromic e-paper.

2. PEG-based IL-GPE.

In 2008, by in situ photocuring, Sun et al. prepared a series of new ionic liquid gel electrolytes based on PEGDA [102]. In addition to the PEGDA monomer and photoinitiator, the formula also contains different amounts of ionic liquid [BMIM][BF_4_] or [BMIM][PF_6_]. These gel electrolytes were used to produce PEDOT-based, poly [3,6-bis(2-(3,4-vinyldioxy)thiophenyl)-N-methylcarbazole] (PBEDOT-NMCz) complementary ECDs. The researchers found that with the increase in ionic liquid content, ECDs exhibit faster switching times, and the coloring and fading times of all ECDs are around 1 s. This is an early attempt to use PEGDA as a polymer matrix.

**Figure 24:**
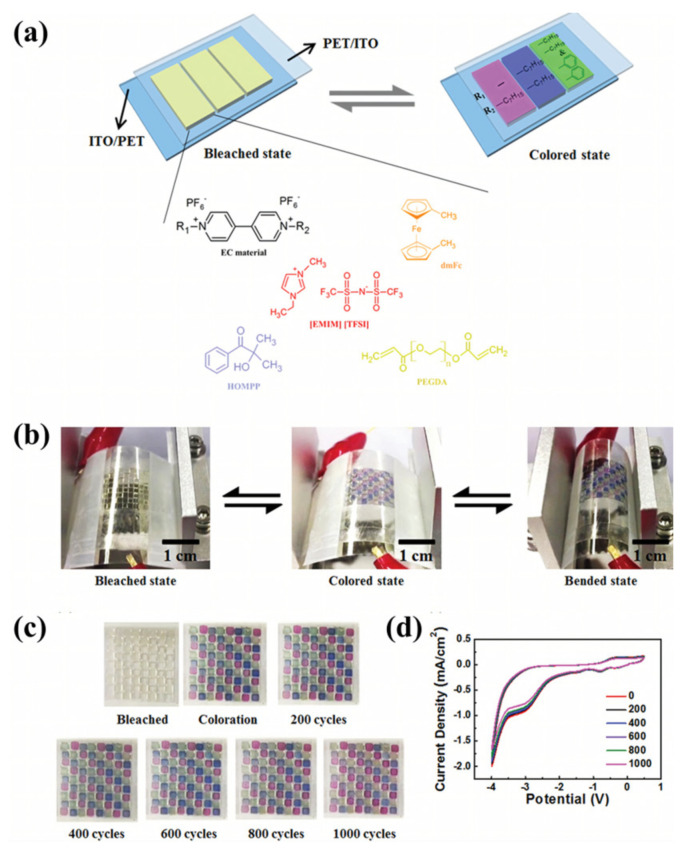
(**a**) Illustration of the chemical structures of the materials forming the EC gels and the basic schematic of an ion gel-based ECD. (**b**) Characteristics of flexible ECDs during dynamic bending tests. (**c**) Camera images and (**d**) CVs of ECDs during the dynamic bending cycles. Camera images in the colored state were captured at −2.5 V [105].

Kim et al. prepared three kinds of EC gels through UV curing technology. In these EC gels, PEGDA crosslinked after UV curing was used as the polymer matrix, [EMIM][TFSI] was used as the plasticizer and conductive ion, and dmFc was used as the redox mediator. MHV, DHV, and DHV-DPV composites were used as electrochromic materials to realize magenta, blue, and green EC gels (Figure 24a) [105]. These three EC gels could be directly sandwiched between two ITO-PET films to form an all-in-one flexible ECD. All three ECDs exhibit high transmittance (>90%) and electrochromic stability, showing stable performance over 3600 s of repeated operation. In addition, the researchers prepared multi-color flexible pixelated ECDs using the mask method, which exhibits excellent mechanical stability even after 1000 bending tests at a curvature radius of 10 mm and outstanding electrochemical stability (after 1000 cycles the current response is almost unchanged) (Figure 24b–d). It should be highlighted that this work represents the first report to achieve multi-color changes and image display through direct patterning of EC materials.

**Table 4:** The performance of polyoxyethylene-based IL gel polymer electrolytes and ECDs.

Electrolyte Membrane				Performance of ECD						
Electrolyte	Conductivity (S/cm)	Transmittance (%)	Thickness (μm)	ECM	Color Changes	t_b_/t_c_ (s)	ΔT (%)	CE (cm^2^/C)	Cycle	Ref.
P(EO)_10_LiTFSI + 0.96 PYR_14_TFSI	1.1 × 10^−4^	/	/	WO_3_	/	/	40–50	/	10^3^	[101]
(PEO)_10_LiTFSI: 25% P_14_TFSI	1.0 × 10^−4^	90%	80	PEDOT PPY	/	1.1/4.0	43	/	10^4^	[103]
VGE	4.27 × 10^−3^	/	200	PProDOT-Me	light blue to dark blue	3.3/2.9	ΔR = 39.4%	76.2	2000	[83]
PEDGA(40%)/ [BMIM][BF_4_] (60%)	3.78~9.49 × 10^−3^	/	/	PEDOT PBEDOT-NMCz	deep blue to yellow transparent	0.4/0.5	/	/	/	[102]
PEGDA/ [EMIM][TFSI]	/	/	60	MHV DHV DHV-DPV	Transparent to magenta, to blue, and to green	31/19 (magenta), 34/20 (blue), 21/39 (green)	/	80.7 (magenta), 81.3 (blue), 54.6 (green)	/	[105]

#### 2.2.3. Polyvinylidene Fluoride-Based IL-GPEs

Polyvinylidene fluoride (PVDF) is a common polymer matrix for gel polymer electrolytes. It is easy to process and possesses good mechanical properties, a high dielectric constant, hydrophobicity, and thermal and chemical stability, making it suitable for the processing and manufacturing of electrolytes [84]. However, due to the high crystallinity of pure PVDF, copolymers with lower crystallinity, such as poly(vinylidene fluoride-co-hexafluoropropylene) (PVDF-HFP) and poly(vinylidene fluoride-trifluoroethylene) (PVDF-TrFE), have been applied in the preparation of electrochemical devices [32,109]. The amorphous regions are beneficial for retaining liquid electrolytes, and the crystalline regions help to maintain mechanical properties and improve the overall performance of polymer electrolytes. Currently, polyvinylidene fluoride-based ionic liquid gel polymer electrolytes have been widely used in the field of electrochromism, and their applications will be discussed below. To elucidate the key performance characteristics of polyvinylidene fluoride-based IL-GPEs and their associated ECDs, we have systematically collated the pertinent data, which are detailed in Table 5 and Table 6 (located at the conclusion of Section 2.2.3).

Electrospinning technology is simple to operate and easy to prepare, and it can effectively combine with different components. This technology can efficiently synthesize composite nanofibers that are thin, highly porous, and flexible. It is a universal and powerful method for preparing one-dimensional nanofibers [110]. The high porosity and thinness of these nanofibers are beneficial for the polymer matrix to absorb electrolytes and transport ions. Therefore, this technology had been widely applied in the preparation of electrolytes.

**Figure 25:**
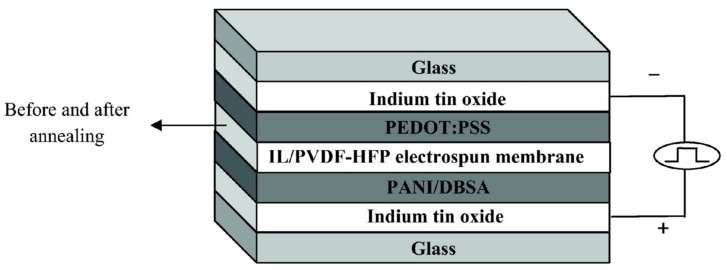
Configuration of the complementary electrochromic devices using the IL/PVDF-HFP electrospun membrane as the electrolyte layer [111].

In 2011, Lu et al. first reported a gel polymer electrolyte using a PVDF-HFP electrospun membrane as the polymer matrix and ionic liquid [BMIM][TFSI] as the plasticizer and electrolyte salt [111]. The thickness of the electrospun film was 33 μm. The ionic conductivity of this gel electrolyte, based on the electrospun membrane, remained essentially unchanged after two days of heat treatment at 60 °C (1.13 × 10^−^³ S/cm after annealing), and the transmittance was greater than 80%. This demonstrated the excellent thermal stability of the gel polymer electrolyte based on the PVDF-HFP electrospun membrane and ionic liquid. The structure of the complementary electrochromic device prepared by the team using PANI and poly(3,4-ethylenedioxythiophene)-poly(4-styrenesulfonic acid) (PEDOT: PSS) (as shown in Figure 25). Under a constant applied potential of ±1.4 V, the device could switch well between the reduced state (yellow-green) and the oxidized state (blue), with a maximum absorbance change of ΔA = 0.242 (at 695 nm). Later, the team carried out extensive related work on the preparation of ionic liquid gel polymer electrolytes based on PVDF-HFP electrospinning membranes. By adjusting the thickness of the electrolyte membrane [112], introducing inorganic fillers [113,114], and incorporating proton conduction [115], the ionic conductivity of the electrolyte was improved, thereby enhancing the performance of ECDs based on PANI-TiO_2_ (as shown in Table 5: properties of IL-GPE and ECD based on polyvinylidene fluoride electrospinning membrane).

**Figure 26:**
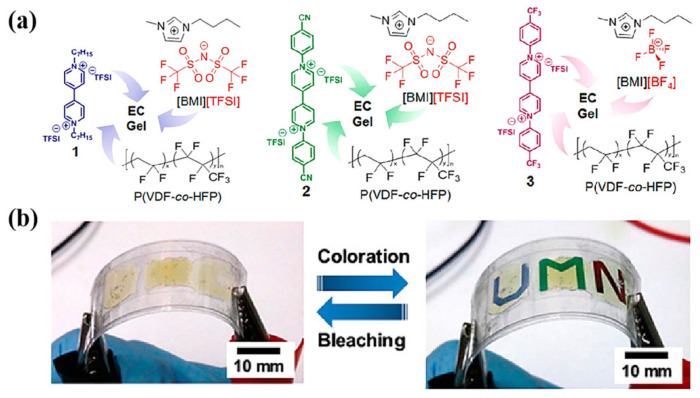
(**a**) Composition diagram of blue, green, and red electrochromic gels (dmFc is not shown). (**b**) Photographs of bleached (at 0.00 V) and colored (at −0.70 V) flexible ECDs with bending [116].

In addition, ionic liquid gel electrolyte based on PVDF can also be made into EC gel to realize an all-in-one electrochromic device. In 2016, Moon et al. prepared three kinds of EC gels based on PVDF-HFP [116]. In these EC gels, PVDF-HFP was used as the polymer matrix, [EMI][TFSI] was used as the electrolyte salt and plasticizer, and HV[TFSI]_2_, CN-PV[TFSI]_2_, and CF_3_-PV[TFSI]_2_ were used as EC materials to realize blue, green, and red, respectively (as shown in Figure 26a). The three EC gels are, respectively, made into three all-in-one ECDs. All three types of ECDs exhibited excellent optical contrast (ΔT = 95%), high coloring efficiency (blue 78 cm^2^/C, green 155 cm^2^/C, and red 81 cm^2^/C), and fast coloring time (blue 11 s, green 18 s, and red 40 s). Furthermore, it is important to note that compared with other ultra-low power displays, such as cholesterol LCD displays with 1 mW/cm^2^, this ECD had extremely low energy consumption (90 μW/cm^2^ for blue, 4 μW/cm^2^ for green, and 32 μW/cm^2^ for red). Finally, the researchers also demonstrated the patterned multi-color flexible ECD prepared with EC gels, which could be electrochromic in the bending state (as shown in Figure 26b). In their follow-up, the team achieved voltage adjustable flexible multi-color ECDs by mixing MHV⁺ and DHV_2_⁺ on this basis [117].

**Figure 27:**
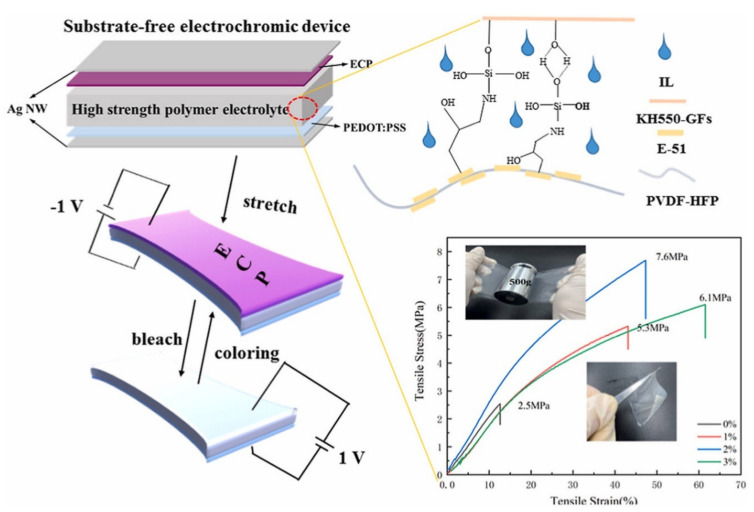
Schematic diagram of ECD using high strength ionic liquid polymer composite electrolyte [118].

In order to obtain PVDF-based gel electrolytes with stronger mechanical strength, in 2022, Zhang’s team prepared a series of high-strength ionic liquid polymer composite electrolytes by introducing glass fibers (GFs) and epoxy resin into PVDF-HFP [118]. In this composite electrolyte, PVDF-HFP was used as the polymer matrix, 1 M LiTFSI/[BMIM][TFSI] solution was used as the electrolyte solution, modified glass fiber KH550-GFs enhance the mechanical properties of the polymer electrolyte, and epoxy resin E-51 was used to improve the interfacial compatibility between GFs and PVDF-HFP (as shown in Figure 27). They studied the transmission spectra, ionic conductivity, and tensile stress–strain of polymer electrolytes made from PVDF-HFP, GFs, and E-51 resins at different ratios. Finally, considering the comprehensive performance of the above aspects, a polymer electrolyte with a PVDF-HFP/E-51 resin mass ratio of 3:1 was used for subsequent assembly and testing. The ECD was assembled with PPRODOT as the working electrode and PPRODOT-BisEDOT as the counter electrode and exhibits a very quick switching speed (t_b_ = 0.5 s, t_c_ = 1.2 s), high coloring efficiency (453 cm^2^/C), and ultra-high electro-chromic stability (with almost no change in optical contrast after 18,000 cycles). In addition, the team also developed a substrate-free ECD based on PPRODOT. This substrate-free ECD exhibits significant color changes, and even after 10,000 compression bending cycles, T_rec_/T_neu_ only undergoes a slight change of about 3% (T_rec_ represents the transmittance of ECD in the recolored state, and Tneu represents the transmittance of ECD in the initial neutral state at a wavelength of 638 nm).

**Table 5:** Properties of IL-GPE and ECD based on polyvinylidene fluoride electrospinning membrane.

Name of Electrospinning Membrane	Thickness (μm)	EC Materials	Conductivity (S/cm)	Tensile Strength (MPa)	Optical Contrast	t_c_/t_b_ (s)	Ref.
PVDF-HFP SCCO_2_	33	PANI/DBSA	1.1 × 10^−3^	6.5 ± 0.4	Δ*A* = 0.23	/	[111]
P(VDF-HFP)/DAP	2	PANI/DBSA	2.6 × 10^−3^	11.4 ± 0.5	Δ*A* = 0.53	/	[112]
PVDF-co-HFP/POSS-OH	/	PANI-TiO_2_	5.2 × 10^−3^	/	ΔT = 71.9%	2.0/2.3	[70]
SiO_2_-on-P(VDF–HFP)	/	PANI-TiO_2_	8.6 × 10^−3^	/	ΔT = 72%	/	[71]
sulfonic acid-grafted P(VDF-HFP)	/	PANI-TiO_2_	6.7 × 10^−3^	/	ΔT = 56%	2.0/2.5	[115]

**Table 6:** Properties of IL-GPE and ECD based on polyvinylidene fluoride.

Electrolyte Membrane				Performance of ECD						
Electrolyte	Conductivity (S/cm)	Transmittance (%)	Thickness (μm)	ECM	Color Changes	t_b_/t_c_ (s)	ΔT(%)	CE (cm^2^/C)	Cycles	Ref.
PVDF/EC-gel	10^−4^~10^−3^	/	60	HV[TFSI]_2_ CN-PV[TFSI]_2_ CF_3_-PV[TFSI]_2_	yellow to blue, green, and red	11/280 (blue) 18/300 (green) 40/180 (red)	>95%	78 (blue) 155 (green) 81 (red)	10^3^	[116]
PVDF-HFP/GFs/E-51	1.75 × 10^−5^	80	40	PPRODOT	blue to puple	1.2/0.5	40	451	18,000	[118]

#### 2.2.4. Polyacrylate-Based IL-GPEs

Polyacrylate-based polymers attracted the attention of many researchers in the field of electrochromism due to their transparency and excellent adhesion. In addition, electrolyte systems based on polyacrylate can be polymerized in situ using corresponding acrylic monomers, which can greatly improve the interface contact between EC electrodes and electrolytes. Such promising properties of polyacrylate-based systems led to significant advancements in electrochromic application. At present, some researchers combined IL with polyacrylic esters to carry out related work on electrolytes [33,119,120,121,122,123,124,125,126,127]. In the following section, the application of gel polymer electrolytes, which contain ionic liquids and are based on polyacrylate polymers, in the field of electrochromism will be discussed. To explain the key performance characteristics of polyacrylate-based IL-GPEs and their associated ECDs, we have summarized the relevant data, which are detailed in Table 7 (located at the conclusion of Section 2.2.4).

**Figure 28:**
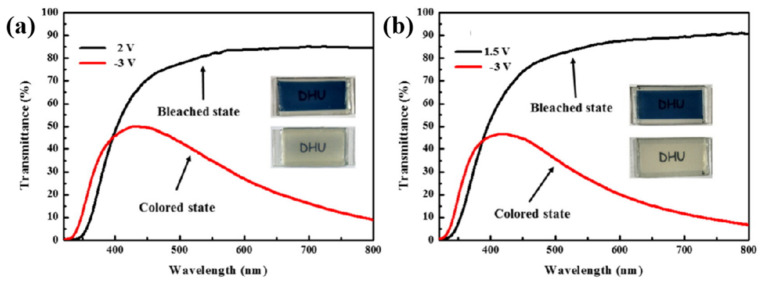
UV–vis transmittance spectra of ECDs with (**a**) PMMA-based electrolyte and (**b**) PMMA–[Emim]BF_4_ composite electrolyte measured at −3 V/2 V and −3 V/1.5 V, respectively. Insets show digital photographs of ECDs [33].

In 2017, Wang’s team prepared a new poly(methyl methacrylate)(PMMA)-based ionic liquid gel electrolyte (PMMA–[Emim] BF_4_) [33] by mixing ionic liquid [EMIM][BF_4_] with a system of PMMA and Li+. PMMA serves as the polymer matrix, with PC acting as the plasticizer and LiClO_4_ along with [EMIM][BF_4_] serving as electrolyte salts in the overall system. PMMA–[Emim] BF_4_ was applied in ECD using WO_3_ as the electrochromic material. Compared with the ECD using PMMA gel electrolyte without ionic liquid (ΔT = 61%, t_b_ = 61.4 s, CE = 33.5 cm^2^/C, and bleaching voltage 2 V), ECD based on PMMA–[Emim] BF_4_ composite electrolyte shows higher optical contrast (ΔT = 71%) (as shown in Figure 28a,b), shorter bleaching time (t_b_ = 41.2 s), lower bleaching voltage (1.5 V), and higher coloring efficiency (55.3 cm^2^/C). The author believes that this improvement is attributed to the higher conductivity (2.9 × 10^−3^ S/cm) of the PMMA–[Emim] BF_4_ composite electrolyte compared with the PMMA gel electrolyte (1.7 × 10^−3^ S/cm).

**Figure 29:**
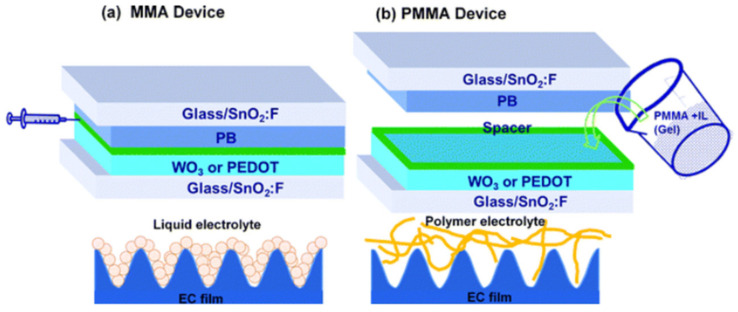
Schematics of the protocols used for device fabrication in (**a**) MMA and (**b**) PMMA-based devices and the corresponding electrode–electrolyte interface in each case [128].

The biggest advantage of polyacrylate-based gel electrolytes is that they can be synthesized by photo or thermal initiation in situ polymerization, which can not only reduce the preparation difficulty of gel electrolytes, but also makes good contact between the electrode and electrolyte interface. There have been previous studies [119,120] on the preparation of electrochromic gels containing ionic liquids through photoinduced in situ polymerization. However, no study had been conducted on the effect of in situ polymerization of gel polymer electrolytes containing ILs on the performance of ECDs. Given the potential benefits of in situ polymerization for electrochromic applications, further exploration was essential, as demonstrated by the following study. In 2012, Deepa et al. [128] prepared polyacrylate-based ionic liquid gels with polymer electrolytes in two ways. One is to inject the MMA monomer containing ionic liquid into ECDs for thermal initiation in situ polymerization (Figure 29a), and the other is to directly inject the prepared PMMA ionic liquid gel electrolyte into ECDs (Figure 29b). The author used PEDOT and WO_3_ as EC materials, with PB as the counter electrode, to prepare four ECDs using the two methods mentioned above: WO_3_-PB (MMA) and PEDOT-PB (MMA) were prepared by injecting the MMA monomer containing ionic liquid into ECD for thermal initiation in situ polymerization, while WO_3_-PB (PMMA) and PEDOT-PB (PMMA) were prepared by directly injecting the prepared PMMA ionic liquid gel electrolyte into ECD. The test results show that WO_3_-PB (MMA), prepared using in situ polymerized polyelectrolyte (Δ Amax = 0.79 a.u., CE = 119 cm^2^/C), shows about four times the absorbance change and twice the coloration efficiency compared with WO_3_-PB (PMMA) directly injected into gel electrolyte (ΔAmax = 0.2 a.u., CE = 54 cm^2^/C). Researchers also found that when PEDOT-PB (MMA) and PEDOT-PB (PMMA) (at −1.5 V) have similar coloring efficiencies, PEDOT-PB (MMA) requires a smaller driving voltage (−0.7 V). In addition, the author also reported the charge transfer resistance of PEDOT-PB ECDs, with the test results showing that the charge transfer resistance of PEDOT-PB (MMA) was 11.5 Ω, while that of PEDOT-PB (PMMA) was 32 Ω. It is believed that the electrolyte polymerized in situ has lower viscosity before polymerization, allowing it to better penetrate into the EC electrode, form better interface contact, and thus reduce charge transfer resistance.

**Figure 30:**
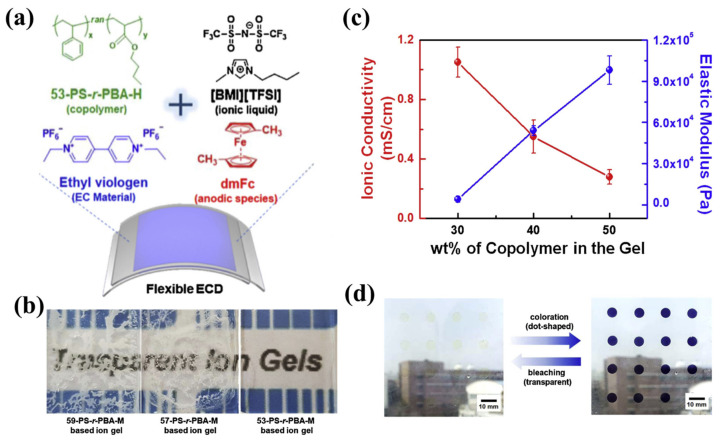
(**a**) Molecular structures of EC gel components. (**b**) Photographs of ion gels based on three PS-r-PBAs having similar molecular weights: 59-PS-r-PBA-M, 57-PS-r-PBA-M, and 53-PS-r-PBA-M, in which 60 wt% of [BMI][TFSI] was included in the gels. (**c**) Plots of ionic conductivity and elastic modulus as a function of wt% of copolymer gelators. All experiments were conducted at 25 °C. (**d**) photographs of coloration/bleaching states of EC smart windows involving dot-shaped EC gel arrays [121].

In 2020, Hong’s team used different proportions of styrene (Sty) and butyl acrylate (BA) to copolymerize a series of poly(styrene-ran-butyl acrylate) copolymers (PS-r-PBAs) [121] through RAFT polymerization. A series of ionic gels was prepared with [BMI][TFSI] as a plasticizer and electrolyte salt (Figure 30a). For the comprehensive consideration of transparency, mechanical properties, and conductivity, the ionic gel containing 60 wt% [BMI][TFSI] and 40 wt% of a specific copolymer (53-PS-r-PBA-H) was applied to the preparation of EC gel (shown as Figure 30b,c). In addition to the above ionic gel components, ethyl viologen (EV^2+^) and dimethyl ferrocene (dmFc) were added to EC gel as electrochromic materials and redox mediators, respectively. The resulting EC gel was then utilized to fabricate a flexible integrated ECD, which demonstrated remarkable performance characteristics. The flexible integrated ECD based on EC gel exhibits high optical contrast (ΔT = 90.1%), switching time (t_c_ = 50 s, t_b_ = 87 s), and high coloring efficiency of 120.8 cm^2^/C. After 1500 cycles, the ΔT retention rate of the flexible ECD was 71.7%, indicating that the EC gel had electrochromic cycle stability. Finally, the author also made a smart window with dot-like EC gel lattice patterns. Because the appearance and disappearance of the dot patterns could be adjusted as needed, this ECD smart window could also be used for bird anti-collision, as the author suggests (Figure 30d).

**Figure 31:**
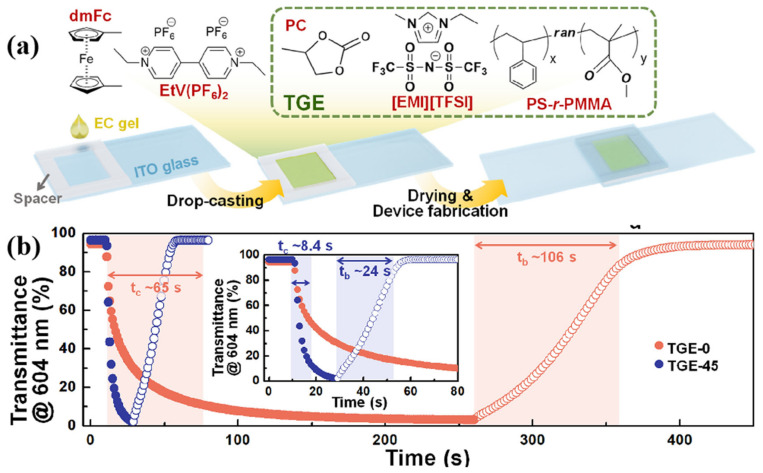
(**a**) Schematic of the fabrication process of ternary gel electrolyte (TGE)-based electrochromic supercapacitors (ECSs) and molecular structures included in the gel. (**b**) Transient transmittance profiles of TGE-0 and TGE-45 ECSs during coloration at +0.9 V and bleaching under a short-circuit condition (i.e., at 0.0 V) [124].

In 2021, Hong’s team prepared a series of ternary gel electrolytes [124] (TGE-0, TGE-20, and TGE-45) composed of copolymer PS-r-PBAs, [EMI][TFSI], and PC (Figure 31a). Among them, TGE-45, which contains 45% PC and [EMI][TFSI] by mass, exhibited the best ion conductivity (5.84 × 10^−3^ S/cm), while TGE-0 (serves as the control group) without PC showed the lowest ion conductivity (2.40 × 10^−3^ S/cm). Therefore, both TGE-0 and TGE-45 were used for further research on all-in-one ECDs. In these ECDs, ethyl viologen (EtV^2+^) and dmFc were used as electrochromic materials and redox mediators, respectively, while [EMI][TFSI] played an ion conducting role. The research results indicate that compared with the ECD based on TGE-0 (t_b_ = 106 s, t_c_ = 65 s), the ECD based on TGE-45 exhibits shorter switching times (t_b_ = 24 s, t_c_ = 8.4 s) (Figure 31b). It is believed that this is because the ternary mixture reduces the overall viscosity of the electrolyte, which is more conducive to the diffusion of EtV2+ (D_EtV2+_ = 1.5 × 10^−11^ cm^2^/s in TGE-0, D_EtV2+_ = 1.38 × 10^−10^ cm^2^/s in TGE-45). In addition, the ECD based on TGE-45 demonstrated high cycling stability, with a ΔT retention ratio of 84% after 2000 cycles.

**Figure 32:**
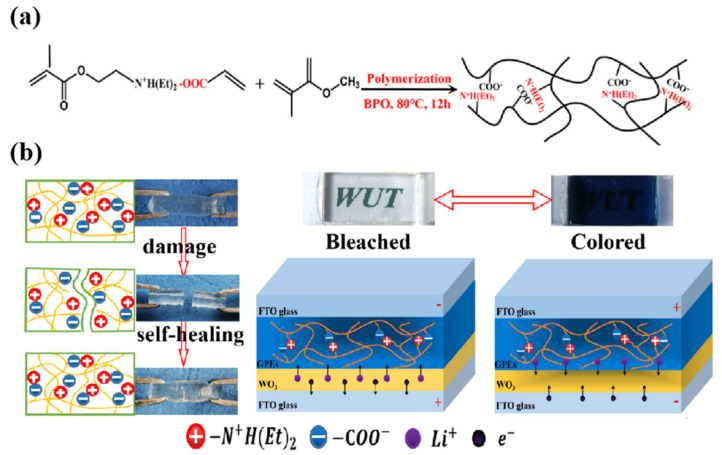
(**a**) Synthesis process of an ionic liquid-based ionically cross-linked gel polymer electrolyte (GPE-ILs). (**b**) Self-healing mechanism of GPE-ILS and schematic diagram of electrochromic device [123].

Additionally, the development of polyacrylate-based electrolytes is also trending towards functionalization. In 2021, Chen et al. prepared a self-healing ionic liquid gel polymer electrolyte (GPE-IL) [123] based on polyacrylate through in situ thermal initiation polymerization (Figure 32a). In this electrolyte, the ion crosslinked polymer network is formed by copolymerization of methyl methacrylate (MMA), acrylic acid (AA), and 2-diethylaminoethyl methacrylate (DEA), with ionic liquid [Emim][TFSI] serving as plasticizer and LiTFSI as electrolyte salt. Due to the presence of the ionic liquid, the gel electrolyte exhibits a high ionic conductivity of 3.29 × 10^−3^ S/cm. A WO_3_-based ECD was prepared using the IL-GPE, which exhibited an optical contrast of 49.9%, a coloring efficiency of 96.2 cm^2^/C, and a fast switching speed with t_b_ = 4 s and t_c_ = 7 s. These outstanding properties of the IL-GPE electrolyte not only enhance the performance of the ECD, but also contribute to its unique self-healing capability, as detailed below. Within the cross-linked polymer networks, due to the electrostatic interaction between -COO^−^ and -N^+^(CH_3_CH_2_)_2_, when GPE-IL is cut and the pieces are placed together again, the network could reconnect to form ionic bonds through electrostatic attraction, thereby exhibiting a self-healing capability (Figure 32b). After self-healing, it could retain 80% of its original tensile strength.

**Figure 33:**
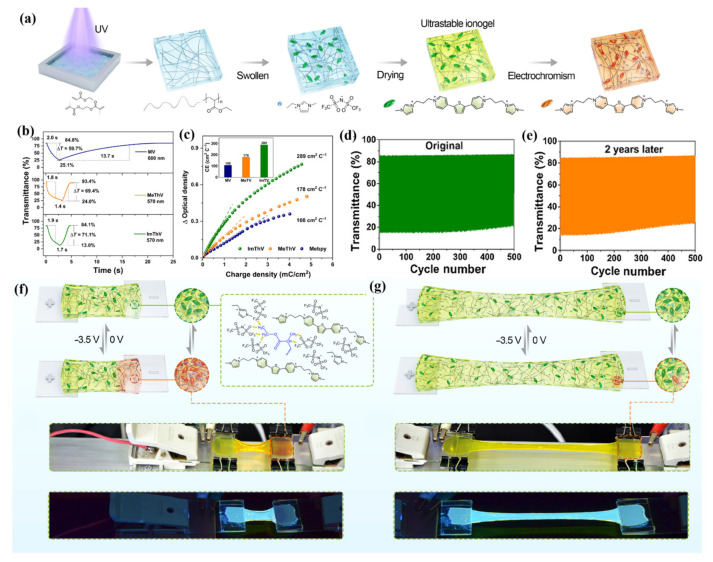
(**a**) Schematic illustration of the fabrication procedure of the ionogel. (**b**) Coloration and bleaching time of [MV][TFSI], [MTV][TFSI], and [ImTV][TFSI]-containing ionogels-based ECDs. (**c**) Plots of the optical density versus charge density and the slope as coloration efficiency (η) for ECDs with EC gel under −3.5 V. (**d**) Freshly made ionogels and (**e**) ionogels stored in ambient atmosphere for 2 years. Illustration and digital pictures of EC properties of stretchable ionogel-based ECD at relaxed (**f**) and stretched state (**g**) [126].

In 2023, He et al. prepared a series of double-network IL-GPE based on polyacrylate ethyl ester for sensing applications [126]. The double-network polymer matrix in the ionic gel was prepared through a two-step process involving photoinitiated polymerization. First, the single network film was prepared using EA monomer and ethylene glycol dimethacrylate (EGDMA) as a crosslinker (Figure 33a). Subsequently, the second network was formed by swelling the initial film and then conducting photoinitiated polymerization. In addition to the polymer matrix, the ionic gel also contains the ionic liquid [EMIM][TFSI] as both a conductive medium and a plasticizer, as well as viologen derivatives for electrochromism and electrofluorescence. In this work, three kinds of ionic gels made of three viologen derivatives, [MV][TFSI], [MTV][TFSI], and [ImTV][TFSI], were prepared. Compared with the other two ionic gels, the ionic gel based on [ImTV][TFSI] shows the best adhesion to the substrate and the shortest coloring time, making it suitable for subsequent tests (Figure 33b,c). The ECD based on [ImTV][TFSI] demonstrated excellent long-term cycling performance, with a ΔT retention rate of 93% after 500 cycles (Figure 33d). Moreover, owing to the nonvolatile nature of the ionic liquid, the ionic gel exhibits robust environmental stability. After two years of storage in air, it maintains stable operation, with a ΔT retention rate of 88% for 500 cycles (Figure 33e). Additionally, the authors further developed a stretchable ECD. With the increase in ionic gel strain levels, the conductivity of the ionic gel shows a downward trend, which weakens the EC and EFC performance of the ECD. At strain levels exceeding 300%, the ionic gel cannot accommodate the EC/EFC phenomenon, thereby reducing the device’s EFC and EC effects (Figure 33f,g). This advanced ionic gel, with its exceptional sensitivity and stability, is highly promising for integration into visible light strain sensors, offering potential benefits in fields such as smart textiles and health monitoring.

**Figure 34:**
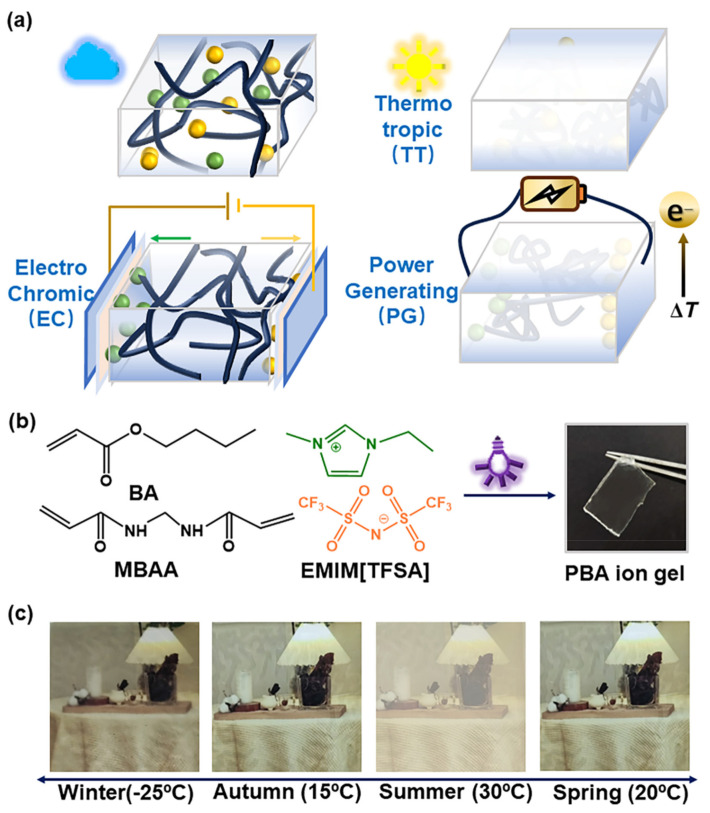
(**a**) An illustration of “all-in-one” smart windows with TT, EC, and PG functions; (**b**) chemical structures of each component to prepare the transparent PBA ion gel via in situ UV polymerization; and (**c**) light transmittance changes in the PBA ion gel-based smart windows at typical temperatures in each season [3].

In 2024, Zhang’s team developed a lower critical solution temperature (LCST) ionic gel based on butyl polyacrylate(PBA) [3]. The PBA ionic gel was prepared by in situ UV polymerization of butyl acrylate (BA) and N,N′-methylenebisacrylamide (MBAA) in ionic liquid [EMIM][TFSI] (Figure 34b). An ‘all-in-one’ smart window was prepared using PEDOT as an electrochromic material and the PBA ionic gel as an electrolyte. In this system, the PBA gel serves the functions of thermochromism and self-powering. The ionic liquid could convert heat energy into electric energy due to its Soret effect [129], thus endowing the ‘all-in-one’ smart window with self-power generation capability. These properties of the PBA ionic gel enable the realization of multiple functionalities in the smart window (Figure 34a). The thermal response temperature range of this smart window is 28–35 °C, and it exhibits significant optical modulation capability, with the visible light transmittance (T_vis_) dropping to 7.5% at 35 °C, effectively blocking visible light (Figure 34c). In terms of electrochromic performance, the smart window achieves an optical contrast of 21%, featuring a coloring time of 7 s and a fading time of 6.8 s. After 100 CV cycles, the smart window demonstrated stable electrochemical performance, with no significant changes in current. This work provides valuable insights and an effective approach for designing multifunctional, intelligent electrochromic smart windows, which hold great promise for reducing building energy consumption and enhancing occupant comfort.

**Table 7:** Performance of polyacrylate-based IL-GPEs and ECDs.

Electrolyte Membrane			Performance of ECD						
Electrolyte	Conductivity (S/cm)	Thickness (μm)	ECM	Color Changes	t_b_/t_c_ (s)	ΔT (%)	CE (cm^2^/C)	Cycling Stability	Ref.
PMMA/LiClO_4_/[Emim]BF_4_	2.9 × 10^−3^	50	WO_3_	/	41.2/61.4	61	55.3	1500 cycles ΔT retention ratio is 71.7%	[33]
MMA (IL-GPE)	1.6 × 10^−4^	/	WO_3_	/	1.7/2.1	0.79 a.u.	119 (at 550 nm)	/	[128]
PS-r-PBAs/[BMI][TFSI]	5.5 × 10^−4^	88	EV	Yellow to purple	50/87	90.1	120.8	1500 cycles ΔT retention ratio is 71.7%	[121]
TGE-45	5.84 × 10^−3^	88	EtV[PF_6_]_2_	Colorless to dark blue	24 s and 8.4 s	94	116.1	2000 cycles ΔT retention ratio is 84%	[124]
P(MMA-AA-DEA)-IL	3.29 × 10^−3^	/	WO_3_	Colorless to blue	4 s and 7 s	49.9	96.2	200 cycles ΔT retention ratio is 70%	[123]
PEA-Ionogel	9 × 10^−4^	1000	[ImTV][TFSI]	light yellow to maroon	1.7 s and 1.7 s	71.7	289	500 cycles ΔT retention ratio is 93%	[126]
LCST-Ionogel	2 × 10^−4^ to 3 × 10^−4^	/	PEDOT	pale blue to dark blue	6.8/7	21.6	/	100 cycles ΔT retention ratio is unknown	[3]

#### 2.2.5. Natural Polymer-Based IL-GPEs

In the research on IL-GPE, in addition to the above common polymer matrix materials, some natural polymer matrix materials have also been used in electrolytes for environmental protection, cost, and other considerations [87,88,89,90,91]. Driven by these considerations, researchers also explored various natural polymer-based IL-GPE for ECDs, pushing forward green and sustainable development in the field.

**Figure 35:**
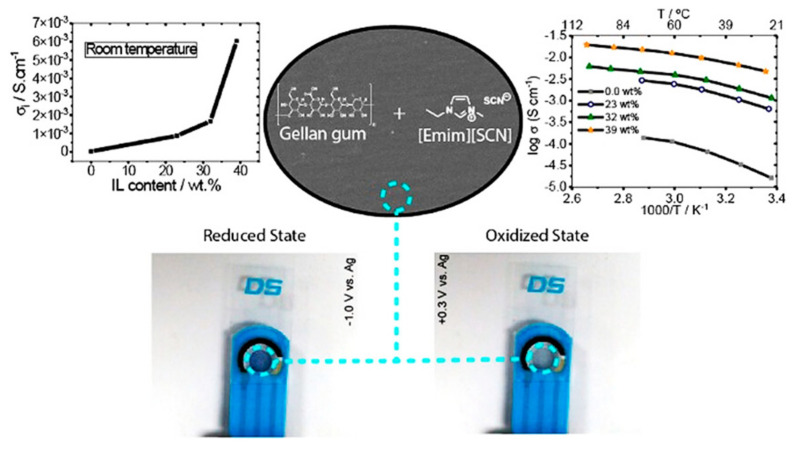
Conductivity of gelled polymer electrolyte containing ionic liquid based on gellan gel and its application in electrochromic devices (The blue circles at the bottom of the figure indicate the location of the electrolyte in the electrochromic device) [89].

In 2022, Alves et al. used the natural polymer gellan gum as the matrix material to develop a new type of IL-GPE (Figure 35) [89]. The chain structure of gellan gum contains a large number of -COOH and -OH groups that easily form complexes with the selected electrolyte salt, thereby facilitating the dissociation of the electrolyte salt and enhancing ion conductivity. In this gel electrolyte, the ionic liquid 1-ethyl-3-methylimidazolium thiocyanate ([Emim][SCN]) and glycerol act as plasticizers, thereby reducing the crystallinity of gellan gum and enhancing conductivity. At 30 °C, the ion conductivity of this environmentally friendly IL-GPE reaches 6.0 × 10^−3^ S/cm. The optical contrast of the ECD prepared by PEDOT: PSS as the working electrode is 30.1% ± 0.1%. Subsequently, the research group reported on agar [90] and carrageenan [91] as polymer matrices to prepare IL-GPE. These works offer promising new avenues for the preparation of high-performance and environmentally friendly ECDs.

#### 2.2.6. Other Polymer-Based IL-GPEs

In addition to the above-mentioned types of polymer electrolytes that have been widely reported, there are also other polymer matrices that have been made into IL-GPE for application in the field of electrochromic technology, such as PU, PVC, PAM, etc., [85,86,130,131,132,133] demonstrating excellent performance. The applications of these materials in ECDs will be elucidated in the following discussion. To clarify the key performance characteristics of these IL-GPEs and their associated ECDs, we have sorted the pertinent data, which are detailed in Table 8 (located at the conclusion of Section 2.2.6).

**Figure 36:**
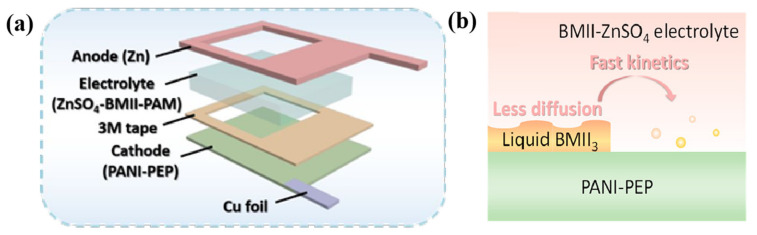
(**a**) Structure diagram of ZPIECBs. (**b**) Schematic of the redox reaction in systems of PANI–PEP/BMII–ZnSO_4_ [85].

Gao et al. prepared a series of hydrogel polymer electrolytes containing ionic liquids, denoted as BMII-ZnSO_4_-PAM [85], and based on PAM with varying BMI contents (0.1 M, 0.3 M, and 0.5 M, respectively). The gel electrolyte was used as an ion conduction layer in a new type of electrochromic battery, namely a zinc polyaniline/iodide electrochromic battery (ZPIECB). In the gel polymer electrolyte, cross-linked polyacrylamide serves as the polymer matrix, ZnSO_4_ functions as the inorganic electrolytic salt, ionic liquid BMII acts as the organic electrolyte salt, and water serves both as the solvent for the electrolyte salts and as a plasticizer for the gel electrolyte. Considering both the conductivity (3.32 × 10^−2^ S/cm) and transparency of the electrolyte, the gel electrolyte 0.3 M BMII-1.5 M ZnSO_4_^−^-PAM, with a BMII content of 0.3 M, was selected for the preparation and testing of ZPIECBs (Figure 36a). The test results show that, in terms of electrochromic performance, ZPIECBs could achieve multi-color changes from light blue (transparent) to green (transparent) to brown (opaque) over a voltage range of 0.3 V to 1.45 V, with a transmittance change (ΔT) of approximately 67.54% at 500 nm. In addition, regarding battery performance, the ZPIECB based on BMII-ZnSO_4_-PAM electrolyte demonstrated superior energy density and coulomb efficiency (1020 μWh/cm^2^, 98.9%) compared with those prepared using the KI-ZnSO_4_-PAM polymer gel electrolyte (182 μWh/cm^2^, 73.2%), which does not contain BMI. This is attributed to the fact that the BMI^+^ ions in BMII could effectively combine with I_3_^+^ to form liquid BMII_3_^+^ (Figure 36b), which not only suppresses the diffusion of I^3–^, but also converts the solid–solid interface between the electrodes into a solid–liquid interface, thereby effectively facilitating interfacial charge transfer and consequently enhancing the electrochemical performance of the battery, resulting in high Coulombic efficiency and rapid reaction kinetics.

**Figure 37:**
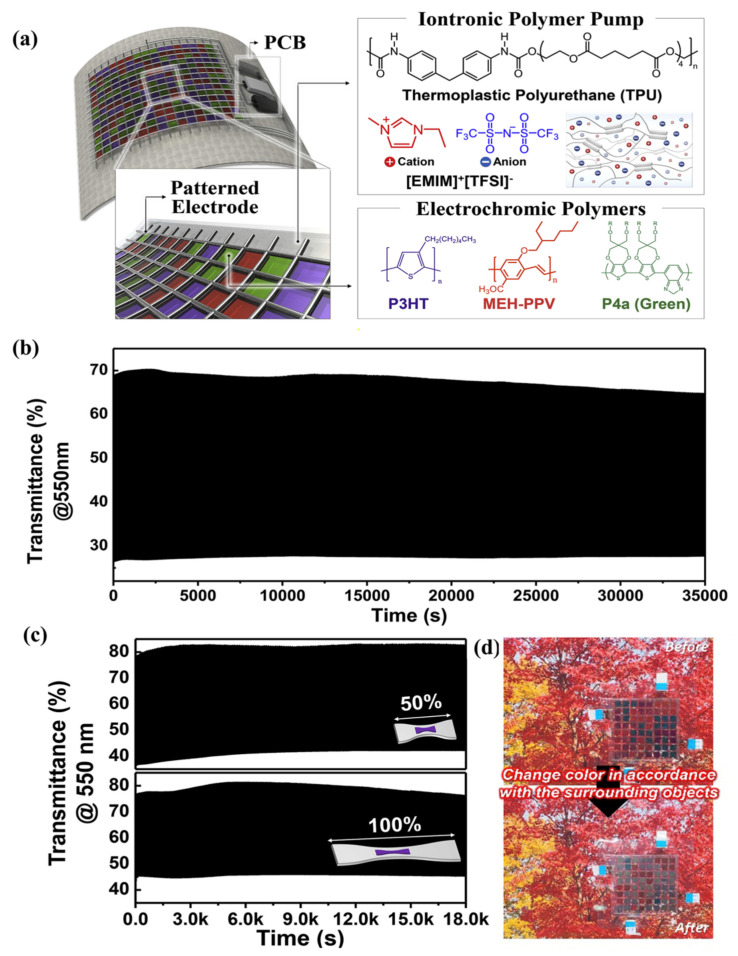
(**a**) Schematic of our DMECS and the chemical structures of its associated components. P3HT, MEH-PPV, and P4a ECPs were used to create purple, orange, and green colors, respectively, while i-TPU ([EMIM]^+^[TFSI]^−^ loaded in TPU polymer chains) was used as a highly flexible solid-state electrolyte. The DMECS was fabricated by sandwiching a multicolor pattern-ECP-incorporated i-TPU film between two flexible electrodes. (**b**) Transient transmittance profile at 550 nm during continuous coloration/bleaching switching cycles of the P3HT ECD. (**c**) Transient transmittance profile of P3HT-incorporated i-TPU active layer in 50% and 100% stretched states. Inset right shows the photographs of P3HT-incorporated i-TPU film in normal and stretched states. (**d**) Real-time photographs of DMECS over various backgrounds showing active camouflage for adaptive camouflage applications [132].

Kim et al. prepared a series of IL-GPEs based on TPU (5i TPU, 10i TPU, 20i TPU, 40i TPU, and 60i TPU) [132]. Ionic liquid [EMIM][TFSI] was used as a plasticizer and electrolyte salt, while TPU was used as a polymer matrix to bind the ionic liquid and provide mechanical properties. Considering the transmittance (93.4%), ionic conductivity (5.4 × 10^−5^ S/cm), and mechanical properties (Young’s modulus 13.4 MPa), 20i TPU was chosen for the manufacture of dynamic multi-color electrochromic skin (DMECS). The team assembled flexible ECDs using three types of EC polymers (P3HT, MEH-PPV, and P4a (green)) that could achieve different color changes (Figure 37a). P3HT-ECD exhibited a fast switching speed (t_b_ = 1.75 s, t_c_ = 1.5 s) and maintained good electrochromic cycling stability even after continuous operation for 35,000 s within the ±3 V potential range (as shown in Figure 37b). Meanwhile, ECDs prepared using MEH-PPV and P4a also exhibit stable long-term cycling behavior. In addition, the team reported on the performance of stretchable ECDs utilizing P3HT. The test results show that the P3HT-DMECS exhibited excellent electrochromic (EC) stability at both 50% and 100% strain, enduring 600 cycles with minimal change in ΔT (as shown in Figure 37c). Finally, the team demonstrated the application of this multi-color ECD for camouflage purposes (as shown in Figure 37d).

**Figure 38:**
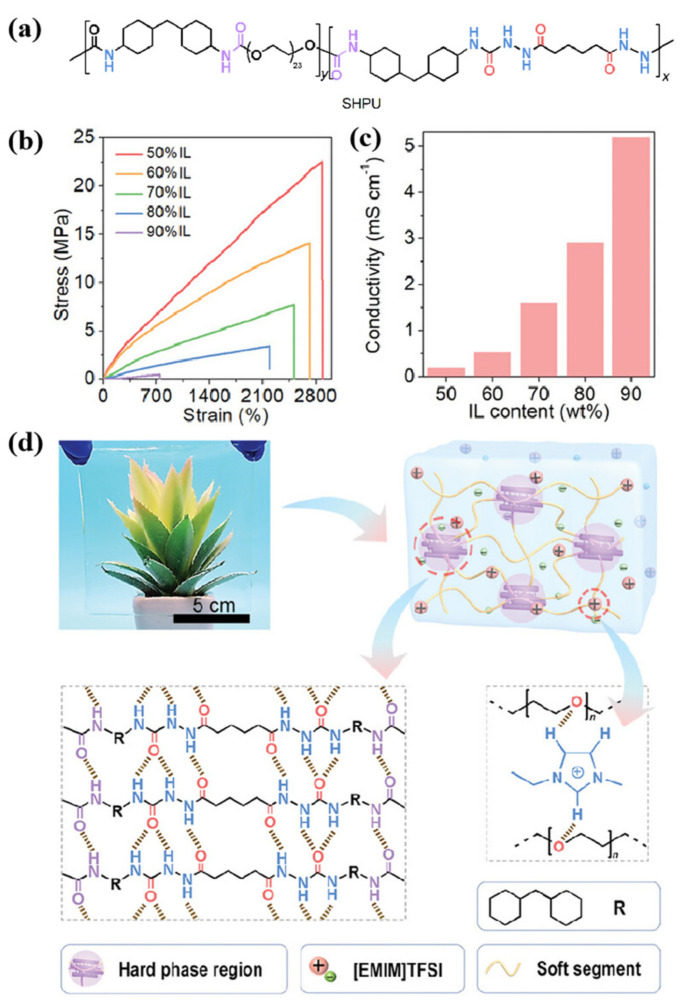
(**a**) Chemical structure of SHPU. (**b**) Stress–strain curves of the SHPU-zIL ionogels. (**c**) Ionic conductivity of the SHPU-zIL ionogels. (**d**) Photograph of the SHPU-60%IL ionogel and schematic illustration of the internal structure of the SHPU-zIL ionogels [86].

Li’s team synthesized a room-temperature self-healing polyurethane, SHPU [86], which contains an acylamino urea structure (Figure 38a). The IL-GPEs containing different [EMIM][TFSI] mass percentages (SHPU-50% IL, SHPU-60% IL, SHPU-70% IL, SHPU-80% IL, and SHPU-90% IL) were prepared with SHPU as the polymer matrix. Although SHPU-90% IL showed the highest ionic conductivity (5.28 × 10^−3^ S/cm), a balance between conductivity and mechanical properties was sought, leading to the selection of SHPU-80% IL for further development into an electrochromic application (Figure 38b,c). SHPU-80% IL was formulated into an EC gel (SHPU-80% IL-EV), containing ethyl viologen bis(hexafluorophosphate) (EV(PF6)2) and hydroxymethyl ferrocene (hmFc), for the assembly and evaluation of ECDs. SHPU-80% IL-EV is sandwiched between two ITO glasses, forming HI-ECD. HI-ECD exhibited a high optical contrast of 94.7% and demonstrated relatively stable electrochromic cycling stability, with a ΔT retention ratio of 93%. Furthermore, it should be highlighted that after cutting the HI-ECD with a glass cutter, the separated parts could be reconnected, and following 18 h of self-repair at room temperature, the cut HI-ECD could achieve a robust connection. This is attributed to the formation of a large number of hydrogen bonds by the acyl amino urea structure in SHPU (Figure 38d), which ensures the excellent self-healing ability of the SHPU-IL at room temperature.

**Figure 39:**
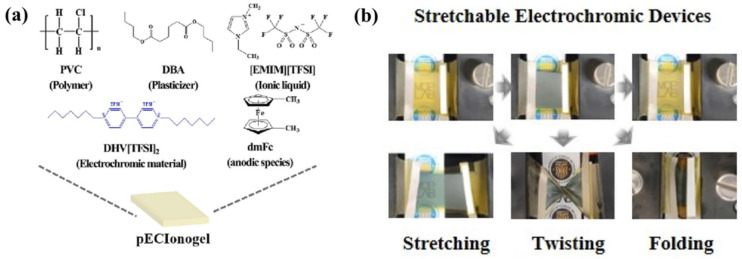
(**a**) Molecular structures of the pECIonogel components: PVC, DBA, EMIMTFSI, DHV[TFSI]_2_, and dmFc. (**b**) Schematic diagram of stretching, twisting, and bending of stretchable electrochromic devices [133].

Bae et al. used polyvinyl chloride (PVC) as the matrix polymer to prepare a series of IL-GPEs, denoted as pECIonogels (-I0.4-M, -I0.8-M, -I1.0-M, -I1.2-M, and -I1.4-M) [133]. Its detailed ingredients (Figure 39a) include dibutyl hexanoic acid (DBA, as a plasticizer), 1-ethyl-3-methylimidazolium bis(trifluoromethanesulfonyl)imide (EMIM TFSI, as an electrolyte salt), DHV[TFSI]_2_ (EC material), and dmFc (redox mediator). These pECIonogels were designed to combine high ionic conductivity with desirable mechanical properties for applications in flexible electronics, and among the pECIonogel series, pECIonogel-I1.2-M exhibited high ionic conductivity (2.8 × 10^−4^ S/cm) and excellent mechanical softening behavior (maximum tensile strain could reach 1664.1%, tensile strength 97.3 kPa, Young’s modulus 8.4 MPa), and was thus used for assembly and testing of subsequent ECDs (Figure 39b). Consequently, flexible ECDs were prepared based on pECIonogel-I1.2-M. The test results show that the flexible ECD prepared based on pECIonogel-I1.2-M demonstrated a high optical contrast of 80.30% and excellent electrochromic cycling stability, with a ΔT retention ratio of 95% after 800 cycles. Furthermore, the team prepared a stretchable ECD based on pECIonogel-I1.2-M. At 0% and 50% tensile strain, the stretchable ECD exhibited optical contrast of 44.24% and 36%, respectively, indicating its responsiveness to mechanical deformation. After 100 cycles of stretching/releasing, the CV curve of the stretchable ECD showed no significant change, thereby demonstrating the electrochemical stability of the stretchable electrochromic device based on pECIonogel-I1.2-M.

**Table 8:** The performance of natural polymer-based IL-GPEs and other polymer-based IL-GPEs in ECDs.

Electrolytes	Conductivity (S/cm)	Transmittance (%)	Thickness (μm)	ECM	Color Changes	t_c_/t_b_ (s)	ΔT (%)	CE (cm^2^/C)	Cycles	Ref.
Gellan Gum-[Emim][SCN]	6.0 × 10^−3^	70–74	120	PEDOT:PSS	/	/	30	/	/	[89]
0.3 M BMII-1.5 M ZnSO_4_-PAM	3.3 × 10^−2^	/	/	PANI-FEP	light blue (transparent)–green (transparent)–brown (opaque)	50/9	67.54	/	/	[85]
20i-TPU	/	93.4	150	P3HT	to purple	1.5/1.75	/	/	/	[132]
SHPU-80%IL	/	/	300	EV(PF_6_)_2_	/	63/255	97.4	106.24	220 cycles ΔT retention ratio93%	[86]
pECIonogel-I1.2-M	2.8 × 10^−4^	90	500	pECIonogel-I1.2-M	yellow to blue	222/178	36	226.67	/	[133]

### 2.3. IL-Based Solid Polymer Electrolyte (IL-SPE)

So far, research on using all solid-state electrolytes (without any liquid components) as EC ionic conductive layers is still in the exploratory stage due to factors such as ion conductivity and transmittance. At present, all solid-state electrolytes reported in the field of electrochromic could be divided into two types: inorganic solid-state electrolytes and solid polymer electrolytes. For example, inorganic solid electrolytes such as Ta_2_O_5_, LiNbO_3_, CaF_2_, etc., Refs [15,16,17,19,134,135,136,137,138,139,140] have been applied in the field of electrochromic. Solid polymer electrolytes based on PVB, PEO, PVA, PVP, etc., [20,22,23,37,141,142,143,144,145,146,147,148] have also been applied in ECDs. In addition to the polymer matrix mentioned above, PIL has also been applied in solid-state polymer electrolytes [24,25,26,27,34,149]. However, to date, there have been limited studies on the application of IL-based solid polymer electrolytes (IL-SPEs) in ECDs. This article briefly analyzes and introduces the relevant reported literature.

**Figure 40:**
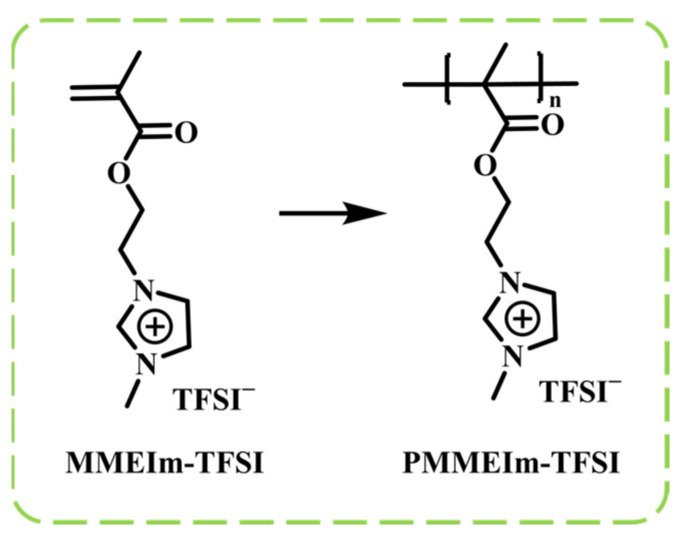
The preparation process of PMMEIm-TFSI.

In 2011, Fu et al. reported on an IL-SPE for application in ECDs. A novel IL (MMEIm TFSI) was first synthesized, followed by the preparation of a PIL, PMMEIm-TFSI [149], via free radical polymerization (Figure 40). After completely dissolving P(MMA-co-VAc), PMMEIm-TFSI, and LiTFSI in THF at various ratios, a series of IL-SPE membranes based on PMMEIm-TFSI could be obtained by casting them into films. The conductivity tests show that the PIL-GPE membrane with 25 wt% P(MMA-co-VAc), 75 wt% PMMEIm-TFSI, and 30 wt% LiTFSI had the highest ion conductivity of 1.78 × 10^−4^ S/cm. Furthermore, the electrolyte membrane exhibited a transparency of 92.1% in the visible light region. Consequently, the membrane was used to fabricate and evaluate ECD, with viologen derivatives serving as the electrochromic layers. The results indicate that this ECD could withstand up to 10^7^ cycles with a response time of about 100 ms.

**Figure 41:**
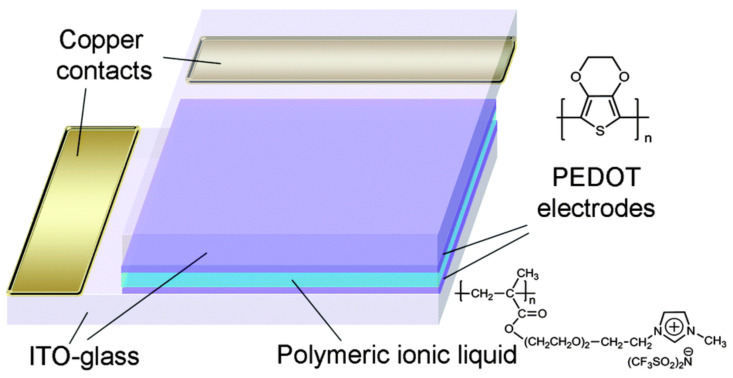
Schematic representation of a symmetrical PEDOT/PIL/PEDOT electrochromic device [34].

In 2014, Alexander’s team developed a solid polymer electrolyte P (MEEMIm TFSI) [34] based on a novel ionic liquid 1-[2-(2-(Methcryloyloxy) ethoxy) ethoxy]-3-methylimidazolium bis (trifluoromethylsul fonyl) imide (MEEMIm TFSI). After casting P (MEEMIm TFSI) into an IL-SPE film, its conductivity was measured to reach 1.0 × 10^−5^ S/cm, indicating that the IL-SPE film had the potential to be used as an electrolyte for ECDs alone. The team applied P (MEEMIm TFSI) as an electrolyte to an all-solid-state ECD using PEDOT as the electrochromic material (Figure 41). The ECD exhibited an optical contrast of 22% at 620 nm, and after 1000 cycles at 0.5–2.5 V, the optical contrast remained unchanged (ΔT remained between 21% and 22%), demonstrating excellent EC cycling stability. Furthermore, it is worth noting that this all-solid-state ECD had a short switching time (3 s for 90% ΔT), which is comparable to the switching times of ECDs using gel or liquid electrolytes (1–8 s) [150,151], and faster than those using other solid polymer electrolytes (4–60 s) [152,153,154].

**Figure 42:**
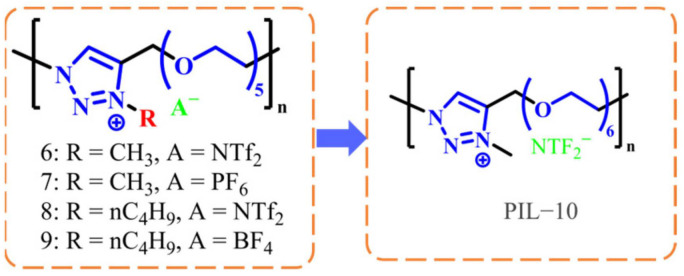
Chemical structure of polymeric ionic liquids (PIL-6, PIL-7, PIL-8, PIL-9, and PIL-10).

In 2018, Puguan et al. synthesized a series of linear structured polyionic liquids PIL6, PIL7, PIL8, PIL9, and PIL10 containing 1, 2, and 3-triazole units (Figure 42) [26,27]. The ionic conductivity test results show that the all-solid poly(ionic liquid) film prepared by PIL10 had the best conductivity, which could reach 1.20 × 10^−4^ S/cm. The PIL10 is therefore used for the construction and testing of all-solid-state ECD. A symmetric all-solid ECD was prepared by combining the all-solid poly(ionic liquid) film (as electrolyte) with PEDOT: PSS(EC active layer). It also shows the characteristics of fast response (t_c_ = 2.5 s, t_b_= 3.2 s) and good EC cycle stability (after 2000 cycles, CR (contrast ratio) decreased from 2.1 to 1.78, only 15%). In addition, although the optical contrast of the device is low (ΔT= 22%), it is higher than ECD (ΔT = 15%) prepared with PEO + Li(CF_3_SO_3_) all-solid electrolyte in devices with the same EC active layer [97].

**Figure 43:**
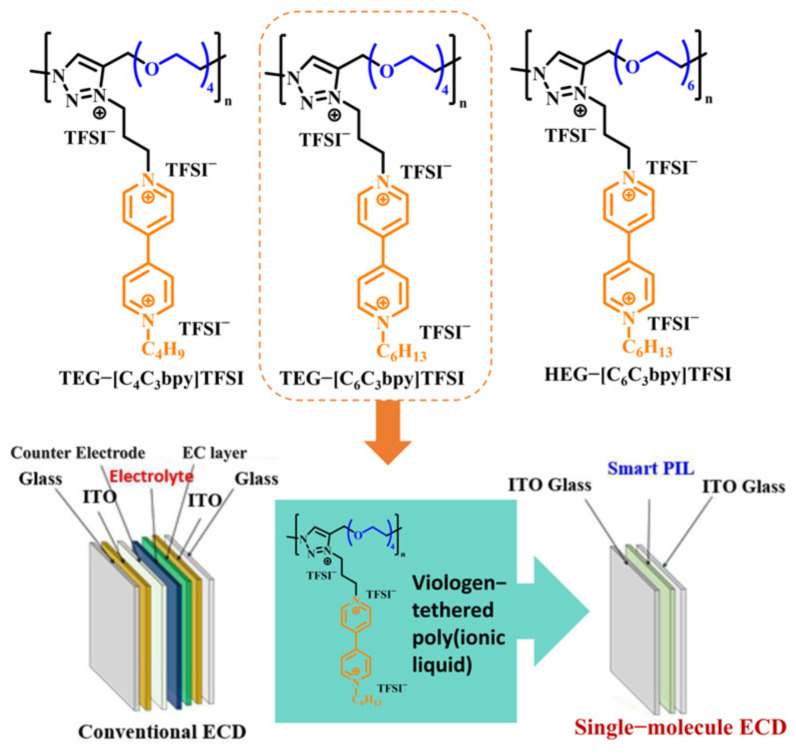
Chemical structures of three types of EC PIL and structural display of single-molecule ECD [25].

In 2019, Puguan et al. introduced a viologen structural unit with electrochromic ability onto the PIL side chain containing triazole groups and prepared a series of novel multifunctional Poly (ionic liquid) TEG-[C_4_C_3_bpy]TFSI, TEG-[C_6_C_3_bpy]TFSI, and HEG-[C_6_C_3_bpy]TFSI that combine electrochromic and ion conductivity (Figure 43) [25]. The authors dissolved the three types of PIL mentioned above in acetonitrile and deposited them onto ITO glass to form all-solid electrolyte films. These Poly (ionic liquid) were then utilized to fabricate all-solid ECDs, aiming to evaluate their electrochromic performance. They combined the films with another piece of ITO glass to construct three types of all-solid integrated ECDs. All three ECDs could achieve color changes from yellow-orange to purple, but in comparison, the ECD based on TEG-[C_6_C_3_bpy] TFSI exhibited the highest transmittance change (ΔT = 21.6%), the highest electrochromic cycle stability with a ΔT retention ratio of 97.22% after 1000 cycles, and exhibited the shortest response time of 14 s.

**Figure 44:**
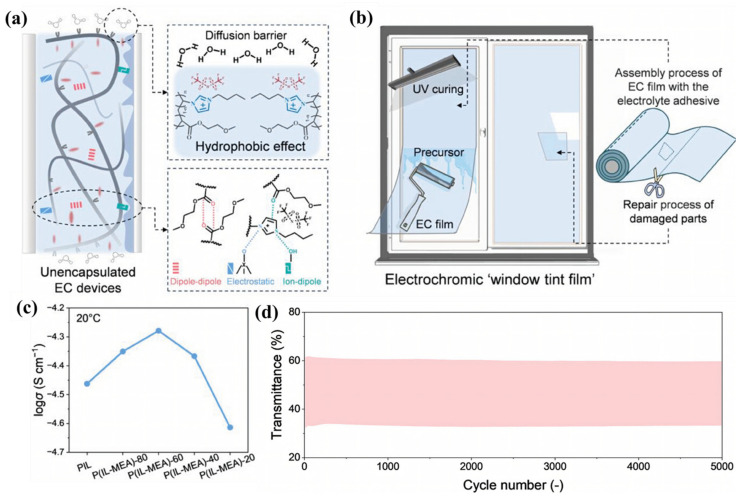
(**a**) Schematic illustration of structural properties for unencapsulated EC devices based on the P(IL-MEA) electrolyte. (**b**) The assembly, installation, and repair process of unencapsulated, removable electrochromic “window tint film”. (**c**) Ionic conductivity of PIL and P(IL-MEA) electrolytes at 20 °C. (**d**) (g) Long-term cyclic test for the EC devices based on P(IL-MEA)^−^ 60 electrolyte [24].

In 2023, Li’s team synthesized a series of novel solid-state PIL electrolytes, P(IL-MEA) [24] (Figure 44a), through in situ photopolymerization. The precursor solution of P(IL-MEA) consists of different mass ratios of 1-butyl-3-vinylimidazolium bis(trifluoromethanesulfonyl)imide VBIm [TFSI] and 2-methoxyethyl acrylate (MEA), as well as succinonitrile (SN) that appears as a solid at room temperature. MEA and VBIm [TFSI] copolymerize to form a polymer matrix that can conduct ions, and SN exists as a plasticizer. P(IL-MEA)-60 had the highest ion conductivity (as shown in Figure 44c), so it was used to construct ECDs with PEDOT: PSS as the electrochromic (EC) layer for subsequent characterization tests. In rigid ECDs, compared with PVDF-based gel electrolytes (after 5000 cycles, ∆T retention rate = 44.6%) and PMMA-based gel electrolytes (after 5000 cycles, ∆T retention rate = 44.6%), ECD based on P(IL-MEA) shows more excellent electrochromic cycle stability (after 5000 cycles, with a ∆T retention rate of 92.5%) (Figure 44d). In addition, in flexible ECDs, after 5000 bending cycles, compared with the PVDF-based gel electrolyte (interfacial separation) and PMMA-based gel electrolyte (electrolyte leakage), P(IL-MEA)-based ECDs exhibit higher mechanical stability (with a ∆T retention rate of 96.3% after 5000 bending cycles). More importantly, based on P(IL-MEA)-60, the research group demonstrated a convenient single-layer EC ‘window tint film’ that simplifies the assembly and maintenance processes for window glass with a conductive layer (Figure 44b). By simply covering the ITO-PET substrate (pre-coated with EC material) with a P(IL-MEA) precursor solution, a smart window can be assembled without the need for packaging. Additionally, if the window tint film is damaged or reaches the end of its service life, it could be quickly repaired or replaced with a new EC film. This research work not only proves that the use of PIL solid polymer electrolyte could improve the electrochromic (EC) cycle life of ECDs, but also provides robust technical support for the convenient assembly, installation, and maintenance of electrochromic smart windows. This work demonstrated the enormous development and application potential of all-solid-state PIL electrolytes in the field of electrochromic smart windows.

Although the application of all-solid-state electrolytes in the field of electrochromism is still in the exploratory phase, it is not difficult to see from the existing research results that the use of solid-state electrolytes especially IL-SPE offers significant advantages in simplifying the device fabrication process, improving the packaging quality, enhancing environmental tolerance, and extending the service life of electrochromic devices (ECDs). To clarify the performance characteristics of IL-SPEs and their corresponding ECDs, we have assembled the relevant data, which are presented in Table 9 (located at the end of Section 2.3).

## 3. Summarization and Prospect

The application of IL-based electrolytes in ECDs demonstrated significant advantages. However, there is currently a lack of comprehensive introduction and analysis reports on the research progress of the application of ionic liquid electrolytes in electrochromic devices in the EC field. Therefore, this article reviews the cutting-edge progress of IL-based electrolytes in the field of electrochromism, summarizes the advantages and characteristics of different types of IL-based electrolytes, and explores the challenges and future development trends they face in the application of ECDs. Based on the summary and induction of existing literature, we found that the use of IL not only effectively improves the electrochromic (EC) cycling stability of ECDs at both room temperature and high temperature, but also broadens the working voltage range of ECDs.

However, low conductivity (high viscosity) and high cost are urgent problems to be solved in the application of IL-based electrolytes in the field of electrochromism (as shown in Table 10). The viscosity of LiClO_4_/organic solvent electrolytes is lower than 3 cP [155], while the viscosity of ionic liquids is much higher than this value. The high viscosity of electrolytes is unfavorable for ion transport, thereby reducing the conductivity of IL-based electrolytes. In addition, the current price of ILs used as electrolytes is relatively high. We researched the prices of several commonly used ionic liquids, which range from USD 500/100 g to USD 3000/100 g. The high cost is a major limitation that restricts the commercial application of ionic liquid electrolytes in ECDs.

We believe that the future development of electrolyte materials based on ILs in the field of EC technology will have the following trends and characteristics:

Towards high-performance electrolytes.

Although the current ionic conductivity of IL-based electrolytes can basically meet the application requirements of ECDs, a higher ionic conductivity will definitely provide a significant boost in achieving ECDs with higher electrochromic performance. How to further improve the ionic conductivity of IL-based electrolytes will be one of the main research directions in this field in the future. In addition, for gel-state and all-solid-state IL-based electrolytes, both the mechanical strength of the electrolyte and its adhesion to the EC electrode should also be considered. Electrolytes with good adhesion and excellent mechanical strength can help to achieve ECDs (especially flexible ECDs) with high electrochromic cycle stability and mechanical stability.

Towards all-solid-state electrolytes.

All solid-state ECDs must be the inevitable trend of future development. Due to their solvent-free nature, the application of all-solid-state electrolytes can significantly enhance both the safety and weather resistance of ECDs. Moreover, all-solid-state ECDs may meet the process requirement of “less packaging or no packaging”, thereby reducing the production cost of ECDs and facilitating their popularization and application. At present, the PIL electrolyte has shown its convenient processing characteristics and great application potential in this aspect and is one of the strong candidates for realizing high-performance all-solid-state ECDs in the future.

Towards low cost.

At present, IL-based electrolytes still face the issue of relatively high cost, which may be one of the current obstacles restricting their further commercial production and application. Currently, as the huge application potential of IL in various fields has been recognized, it can be predicted that with the development of new ionic liquid materials and the maturity of large-scale production technology for ionic liquid materials, cost will no longer be a barrier to its application in the future.

Towards functionalization and intelligence.

With the increasing research on the application of ECDs in military camouflage, biosensors, smart windows, and other fields, IL-based electrolytes are expected to become more functional and intelligent in the future. Currently, some researchers already carried out related work focusing on functionality and intelligence, such as strain response, temperature response, and self-powering. As research in this field continues to deepen, more innovative applications of ECDs based on IL-based electrolytes will undoubtedly emerge in the future.

Towards green and sustainable development.

Green and sustainable development has been the concern of many countries around the world. Therefore, ECD electrolytes should also develop in this direction in the future. Due to its extremely low vapor pressure, IL will not volatilize into the atmosphere and has been considered as a green solvent. Currently, there have been some reports on natural polymer-based IL-gel polymer electrolytes (IL-GPEs), but the number of such reports is limited. In the future, more natural polymer-based IL-gel polymer electrolytes (IL-GPEs) will be widely studied and applied in ECDs.

## Figures and Tables

**Table 2:** The performance of IL liquid electrolytes and electrochromic devices (ECDs).

Electrolytes	Conductivity (S/cm)	ECM	Color Changes	t_c_/t_b_ (s)	ΔT(%)	CE (cm^2^/C)	Cycle	Ref.
[BMIM][BF_4_]	/	PANI	transparent yellow to dark blue	1 ms	63	/	10^6^	[42]
[EMIM][TFSI]	6.37 × 10^−4^	PEDOT: PSS	Light blue to dark blue	12.0/4.0	36	/	/	[68]
BMPyr (NTf)_2_	7.8 × 10^−4^	polyFe	transparent to blueish-violet	12.0/20.5	51	254.5	More than 300 cycles	[43]
[BMIM][BF_4_]	1.81 × 10^−3^	Mg-PDI	red to purple	9.2/0.6	42	305	200 cycles ΔT retention rate 95.8%	[29]
[C_1_C_5_O_2_bpy][NTf_2_]_2_	/	/	yellow to blue	/	/	/	/	[75]
[EV][(NTf)_2_]_2_ [BIMI][FcNTf]	/	[EV][(NTf)_2_]_2_	to blue	9.5/5.5	38.8	113.7	10^3^ cycles ΔT retention rate 94.8%	[78]
[FcC_11_VC_1_][TFSI]_2_	/	[FcC_11_VC_1_][TFSI]_2_	light brown to dark purple	/	/	70	10^4^ cycles	[71]
[C_4_VC_7_][TFSI]_2_ [FcC_6_ImC_1_][TFSI]	/	[C_4_VC_7_][TFSI]_2_	/	/	/	91.4	10^4^ cycles	[79]
[C_6_SVC_6_][TFSI]_2_ [FcC_11_ImC_1_][TFSI]	/	[C_6_SVC_6_][TFSI]_2_	blue to yellow	15.0/23.4	63.2	80.3	3000 cycles ΔT retention rate 98.2%	[80]
Vio-IL Fc-IL	1.4 × 10^−5^ Vio-IL 5.6 × 10^−5^ Fc-IL	Vio-IL	transparent to blue-violet	13.3/3.7	79.4	/	10^6^	[72]
TILBF_4_	1.9 × 10^−2^	PProDOT-Et_2_	light blue to deep blue	3.6/4.0	62.2	983.0	/	[81]
RIL2 (in [BMIM][TFSI])	2.0 × 10^−3^	PRBR	transparent to deep blue–purple	/	>85	/	More than 300 cycles	[82]

**Table 9:** The performance of partial solid polymer electrolytes and ECDs.

Electrolytes	Conductivity (S/cm)	Transmittance (%)	Thickness (μm)	ECM	Color Changes	t_b_/t_c_ (s)	ΔT (%)	CE (cm^2^/C)	Cycle	Ref.
PVB + LiClO_4_	1.78 × 10^−4^	>90	400	WO_3_	/	14.47/17.30	70	/	20,000	[143]
PVA + LiCH_3_COO	1.593 × 10^−^¹¹	90	/	WO_3_	/	0.12/0.1	/	/	150	[146]
PVP-LiClO_4_	/	/	300	WO_3_	Transparent to dark blue	3.36/3.88	/	/	100	[148]
p(TMC)/PEO/LiClO_4_	1.33 × 10^−6^	/	150	PEDOT	light blue to dark blue	Long cycle times (>600 s)	22	/	/	[20]
P(MMA-Vac)/PMMEIm-TFSI/LiTFSI	1.78 × 10^−4^	92.1	/	viologen derivative	/	0.1	/	/	10^7^	[149]
P(MEEMIm TFSI)	1.0 × 10^−5^	/	0.5	PEDOT	light blue to dark blue	3/3	22	390	10^3^	[34]
PIL-6	1.16 × 10^−4^	/	206	PEDOT: PSS	Transparent to blue	11.8/4.7	18	356	/	[23]
PIL-10	1.20 × 10^−4^	/	24	PEDOT: PSS	pale blue to dark blue	3.2/2.5 s	22	/	2000	[24]
TEG-[C_6_C_3_bpy]TFSI	/	/	/	TEG-[C_6_C_3_bpy]TFSI	yellow-orange to purple	14 s	21.6	76.8	10^3^	[25]
P(IL-MEA)	/	92%	200	PEDOT: PSS	/	6.5/1.5 s	/	1021	5000	[24]

**Table 10:** The prices, conductivity, and viscosity of several common ionic liquids.

Brand	Product Name	η (cP)	Window (V)	Conductivity (S/cm)	Price (USD/100 g)
Sigma-Aldrich (St. Louis, MO, USA)	[EMIm][TFSI]	37 [156]	4.3 [157]	8.6 × 10^−4^ [157]	530
	[EMIm][BF_4_]	41 [156]	4.3 [157]	1.38 × 10^−3^ [157]	1337
	[BMIm][TFSI]	52 [157]	4.76 [157]	4 × 10^−3^ [158]	530
	[BMIm][BF_4_]	112 [156]	6.1 [157]	4 × 10^−4^ [159]	1200
	[BMIm][PF_6_]	216 [156]	5 [157]	1.59 × 10^−4^ [159]	2765

## Data Availability

Data are available in the source publications listed in the bibliography.

## Abbreviations

The following abbreviations are used in this manuscript:

EC	Electrochromism
ECD	Electrochromic device
CE	Coloration Efficiency
IL	Ionic liquid
PIL	Polymeric ionic liquid
IL-GPE	Ionic liquid-based gel polymer electrolyte
IL-SPE	Ionic liquid-based solid polymer electrolyte
ITO	Indium Tin Oxide
FTO	Fluorine-doped Tin Oxide
ATO	Antimony-doped Tin Oxide
PET	Polyethylene Terephthalate
PB	Prussian Blue
Fc	Ferrocene
dmFc	Dimethyl Ferrocene
PANI	Polyaniline
PEDOT	Poly(3,4-ethylenedioxythiophene)
PEDOT:PSS	Poly(3,4-ethylenedioxythiophene):Polystyrene Sulfonate
TEMPO	2,2,6,6-Tetramethylpiperidine 1-oxyl
[AMIM][TFSI]	1-Aminomethyl-3-methylimidazolium bis(trifluoromethylsulfonyl)imide
[BMIM][BF_4_]	1-Butyl-3-methylimidazolium tetrafluoroborate
[BMIM][Br]	1-Butyl-3-methylimidazolium bromide
[BMIM][PF_6_]	1-Butyl-3-methylimidazolium hexafluorophosphate
[BMIm][NTf]_2_	1-Butyl-3-methylimidazolium bis(trifluoromethylsulfonyl)imide
[BVIm][Br]	1-Butyl-2-vinylimidazolium bromide
[EMIM][BF_4_]	1-Ethyl-3-methylimidazolium tetrafluoroborate
[EMIM][Br]	1-Ethyl-3-methylimidazolium bromide
[EMIM][SCN]	1-Ethyl-3-methylimidazolium thiocyanate
[EMIM][TFSI]	1-Ethyl-3-methylimidazolium bis(trifluoromethylsulfonyl)imide
[emim][Br]	1-Ethyl-3-methylimidazolium bromide
[ViEtIm][Br]	1-Vinyl-2-ethylimidazolium bromide
[ViEtIm][Tf_2_N]	1-Vinyl-2-ethylimidazolium bis(trifluoromethylsulfonyl)imide
VBIMTFSI	Vinyl-1-butyl-3-methylimidazolium bis(trifluoromethylsulfonyl)imide

## References

[1] Mortimer R.J. (1997). Electrochromic materials. Chem. Soc. Rev..

[2] Deb S.K. (1969). A novel electrophotographic system. Appl. Opt..

[3] Ma Y., Wang Y., Zhou J., Lan Y., Jiang S., Ge Y., Tan S., Zhang S., Wang C., Wu Y. (2024). LCST ion gels fabricating “all-in-one” smart windows: Thermotropic, electrochromic and power-generating. Mater. Horiz..

[4] Shao Z., Huang A., Cao C., Ji X., Hu W., Luo H., Bell J., Jin P., Yang R., Cao X. (2024). Tri-band electrochromic smart window for energy savings in buildings. Nat. Sustain..

[5] Riganti M., Li Castri G., Serra V., Manca M., Favoino F. (2025). Energy saving potential of advanced dual-band electrochromic smart windows for office integration. Energy Build..

[6] Cai G., Wang J., Lee P.S. (2016). Next-Generation Multifunctional Electrochromic Devices. Acc. Chem. Res..

[7] Li W., Bai T., Fu G., Zhang Q., Liu J., Wang H., Sun Y., Yan H. (2022). Progress and challenges in flexible electrochromic devices. Sol. Energy Mater. Sol. Cells.

[8] Fu H., Zhang L., Dong Y., Zhang C., Li W. (2023). Recent advances in electrochromic materials and devices for camouflage applications. Mater. Chem. Front..

[9] Shen W., Li G. (2023). Recent Progress in Liquid Crystal-Based Smart Windows: Materials, Structures, and Design. Laser Photonics Rev..

[10] Meng H. (2021). Introduction. Organic Electronics for Electrochromic Materials and Devices.

[11] Wu W., Guo S., Bian J., He X., Li H., Li J. (2024). Viologen-based flexible electrochromic devices. J. Energy Chem..

[12] Gu C., Jia A.-B., Zhang Y.-M., Zhang S.X.-A. (2022). Emerging Electrochromic Materials and Devices for Future Displays. Chem. Rev..

[13] Thakur V.K., Ding G., Ma J., Lee P.S., Lu X. (2012). Hybrid Materials and Polymer Electrolytes for Electrochromic Device Applications. Adv. Mater..

[14] Patel K.J., Bhatt G.G., Ray J.R., Suryavanshi P., Panchal C.J. (2017). All-inorganic solid-state electrochromic devices: A review. J. Solid State Electrochem..

[15] Zhao Y., Zhang X., Chen X., Li W., Li Z., Chen M., Sun W., Zhao J., Li Y. (2021). All-solid-state electrochromic devices based on the LiAlSiO4 electrolyte. Mater. Lett..

[16] Chen X., Zhang H., Li W., Xiao Y., Zhang X., Li Y. (2022). CaF2: A novel electrolyte for all solid-state electrochromic devices. Environ. Sci. Ecotechnol..

[17] Chen X., Zhang H., Li W., Xiao Y., Ge Z., Li Y., Zhang X. (2022). Electro-optical performance of all solid state electrochromic devices with NaF electrolytes. Mater. Lett..

[18] Liu R., Ren Y., Hou C., Wang J., Wang Y., Tang K., Zhao G. (2024). Preparation of all-solid-state electrochromic devices based on li+-contained phosphate electrolyte. Ceram. Int..

[19] Wang J., Wang X., Zhang C., Lin S., Dai M., Wang H., Xie S., Shi Q. (2023). Influence of N-doped aluminosilicate as an electrolyte on the properties of all-solid-state electrochromic devices. Sol. Energy Mater. Sol. Cells.

[20] Barbosa P.C., Rodrigues L.C., Silva M.M., Smith M.J., Parola A.J., Pina F., Pinheiro C. (2010). Solid-state electrochromic devices using pTMC/PEO blends as polymer electrolytes. Electrochim. Acta.

[21] Lim B.H., Kim J.M., Nguyen V.-T., Kim H., Park C.W., Lee J.K., Lee C.-H., Yoo J., Min B.K., Kim S.K. (2023). Functionalized methyl cellulose/LiClO4 composite as an environmentally friendly quasi-solid polymer electrolyte for solid-state electrochromic devices and cellulose-based supercapacitors. Mater. Today Energy.

[22] Kiristi M., Bozduman F., Gulec A., Teke E., Oksuz L., Oksuz A.U., Deligöz H. (2014). Complementary all solid state electrochromic devices using carboxymethyl cellulose based electrolytes. J. Macromol. Sci. Part A.

[23] Yang Jeong C., Kubota T., Chotsuwan C., Wungpornpaiboon V., Tajima K. (2021). All-solid-state electrochromic device using polymer electrolytes with a wet-coated electrochromic layer. J. Electroanal. Chem..

[24] Wu X., Bai Z., Bao B., Zhang Q., Jiang W., Li Y., Hou C., Li K., Wang H. (2023). A Lithium-Salt-Free, Hydrophobic, Solid-State Poly(Ionic Liquid) Electrolyte Enables Rapid Assembly of Unencapsulated, Removable Electrochromic “Window Tint Film”. Adv. Funct. Mater..

[25] Puguan J.M.C., Kim H. (2019). A switchable single-molecule electrochromic device derived from a viologen-tethered triazolium-based poly(ionic liquid). J. Mater. Chem. A.

[26] Puguan J.M.C., Jadhav A.R., Boton L.B., Kim H. (2018). Fast-switching all-solid state electrochromic device having main-chain 1,2,3-triazolium-based polyelectrolyte with extended oxyethylene spacer obtained via click chemistry. Sol. Energy Mater. Sol. Cells.

[27] Puguan J.M.C., Boton L.B., Kim H. (2018). Triazole-based ionene exhibiting tunable structure and ionic conductivity obtained via cycloaddition reaction: A new polyelectrolyte for electrochromic devices. Sol. Energy Mater. Sol. Cells.

[28] Wei Y., Chen M., Liu W., Li L., Yan Y. (2017). Electrochemical investigation of electrochromic devices based on NiO and WO3 films using different lithium salts electrolytes. Electrochim. Acta.

[29] Lu Z., Li R., Ping L., Bai Z., Li K., Zhang Q., Hou C., Li Y., Jin W., Ling X. (2022). Ultra-stable ionic-liquid-based electrochromism enabled by metal-organic frameworks. Cell Rep. Phys. Sci..

[30] Tan C., Hu Z., Guo Z., Cui Z., Bai L., Wu X., Huang C., Su W. (2024). In-situ-selective-UV crosslinking fabrication of solid liquid host guest electrolyte: A facile one-step method realizing highly flexible electrochromic device. Nano Res..

[31] Cai H., Chen Z., Guo S., Ma D., Wang J. (2023). Polyacrylamide gel electrolyte for high-performance quasi-solid-state electrochromic devices. Sol. Energy Mater. Sol. Cells.

[32] Li W., Bai T., Zhang Q., Liu J., Zhou K., Wang H. (2023). A highly transparent ion conducting film enabling a visual electrochromic battery. J. Mater. Chem. C.

[33] Tang Q., Li H., Yue Y., Zhang Q., Wang H., Li Y., Chen P. (2017). 1-Ethyl-3-methylimidazolium tetrafluoroborate-doped high ionic conductivity gel electrolytes with reduced anodic reaction potentials for electrochromic devices. Mater. Des..

[34] Shaplov A.S., Ponkratov D.O., Aubert P.-H., Lozinskaya E.I., Plesse C., Vidal F., Vygodskii Y.S. (2014). A first truly all-solid state organic electrochromic device based on polymeric ionic liquids. Chem. Commun..

[35] Oukassi S., Giroud-Garampon C., Dubarry C., Ducros C., Salot R. (2016). All inorganic thin film electrochromic device using LiPON as the ion conductor. Sol. Energy Mater. Sol. Cells.

[36] Zhu Y., Xie L., Chang T., Bell J., Huang A., Jin P., Bao S. (2019). High performance all-solid-state electrochromic device based on LixNiOy layer with gradient Li distribution. Electrochim. Acta.

[37] Wang W., Guo S., Feng F., Li Q., Cai H., Rougier A., Ma D., Wang J. (2024). Research Progress in Polymer Electrolytes for Electrochromic Devices. Polym. Rev..

[38] Saha M., Kumar A., Kanaoujiya R., Behera K., Trivedi S. (2024). A Comprehensive Review of Novel Emerging Electrolytes for Supercapacitors: Aqueous and Organic Electrolytes Versus Ionic Liquid-Based Electrolytes. Energy Fuels.

[39] Hu X., Wang Y., Feng X., Wang L., Ouyang M., Zhang Q. (2025). Thermal stability of ionic liquids for lithium-ion batteries: A review. Renew. Sustain. Energy Rev..

[40] Xue Z., Qin L., Jiang J., Mu T., Gao G. (2018). Thermal, electrochemical and radiolytic stabilities of ionic liquids. Phys. Chem. Chem. Phys..

[41] Wang T., Luo H., Bai Y., Li J., Belharouak I., Dai S. (2020). Direct Recycling of Spent NCM Cathodes through Ionothermal Lithiation. Adv. Energy Mater..

[42] Lu W., Fadeev A.G., Qi B., Smela E., Mattes B.R., Ding J., Spinks G.M., Mazurkiewicz J., Zhou D., Wallace G.G. (2002). Use of Ionic Liquids for π-Conjugated Polymer Electrochemical Devices. Science.

[43] Mondal S., Yoshida T., Higuchi M. (2019). Electrochromic devices using Fe(II)-based metallo-supramolecular polymer: Introduction of ionic liquid as electrolyte to enhance the thermal stability. J. Soc. Inf. Disp..

[44] Lu H.-C., Kao S.-Y., Yu H.-F., Chang T.-H., Kung C.-W., Ho K.-C. (2016). Achieving Low-Energy Driven Viologens-Based Electrochromic Devices Utilizing Polymeric Ionic Liquids. ACS Appl. Mater. Interfaces.

[45] Yu H.-F., Li C.-T., Ho K.-C. (2022). Stable viologen-based electrochromic devices: Control of Coulombic interaction using multi-functional polymeric ionic liquid membranes. Sol. Energy Mater. Sol. Cells.

[46] Wang Y.-L., Li B., Laaksonen A. (2021). Coarse-grained simulations of ionic liquid materials: From monomeric ionic liquids to ionic liquid crystals and polymeric ionic liquids. Phys. Chem. Chem. Phys..

[47] Oke E.A., Oluyinka O.A., Afolabi S.D., Ibe K.K., Raheem S.A. (2023). Latest insights on technologies for halides and halogenated compounds extraction/abatement from water and wastewater: Challenges and future perspectives. J. Water Process Eng..

[48] Zaidi S.S.H., Li X. (2023). Li–O2/Air Batteries Using Ionic Liquids—A Comprehensive Review. Adv. Energy Mater..

[49] Khan A.S., Ibrahim T.H., Jabbar N.A., Khamis M.I., Nancarrow P., Mjalli F.S. (2021). Ionic liquids and deep eutectic solvents for the recovery of phenolic compounds: Effect of ionic liquids structure and process parameters. RSC Adv..

[50] Oke E.A., Ijardar S.P. (2021). Insights into the separation of metals, dyes and pesticides using ionic liquid based aqueous biphasic systems. J. Mol. Liq..

[51] Qian W., Texter J., Yan F. (2017). Frontiers in poly(ionic liquid)s: Syntheses and applications. Chem. Soc. Rev..

[52] Md Moshikur R., Goto M. (2023). Pharmaceutical Applications of Ionic Liquids: A Personal Account. Chem. Rec..

[53] Wang X., Hu X., Zhang D., Zhang Y., Xu H., Sun Y., Gu X., Luo J., Gao B. (2024). Environmental applications and toxicity of ionic liquids. J. Environ. Chem. Eng..

[54] Liu S., Wu Y., Jiang L., Xie W., Davis B., Wang M., Zhang L., Liu Y., Xing S., Dickey M.D. (2024). Highly Stretchable, Tissue-like Ag Nanowire-Enhanced Ionogel Nanocomposites as an Ionogel-Based Wearable Sensor for Body Motion Monitoring. ACS Appl. Mater. Interfaces.

[55] Wang Z., Si Y., Zhao C., Yu D., Wang W., Sun G. (2019). Flexible and Washable Poly(Ionic Liquid) Nanofibrous Membrane with Moisture Proof Pressure Sensing for Real-Life Wearable Electronics. ACS Appl. Mater. Interfaces.

[56] Chen C., Ying W.B., Li J., Kong Z., Li F., Hu H., Tian Y., Kim D.H., Zhang R., Zhu J. (2022). A Self-Healing and Ionic Liquid Affiliative Polyurethane toward a Piezo 2 Protein Inspired Ionic Skin. Adv. Funct. Mater..

[57] Cai Y., Hou Y., Lu Y., Zhang Q., Yan Z., Chen J. (2023). Ionic Liquid Electrolyte with Weak Solvating Molecule Regulation for Stable Li Deposition in High-Performance Li-O_2_ Batteries. Angew. Chem. Int. Ed..

[58] Liu K., Wang Z., Shi L., Jungsuttiwong S., Yuan S. (2021). Ionic liquids for high performance lithium metal batteries. J. Energy Chem..

[59] Francis C.F.J., Kyratzis I.L., Best A.S. (2020). Lithium-Ion Battery Separators for Ionic-Liquid Electrolytes: A Review. Adv. Mater..

[60] Wang Y., Xue K., Zhang X., Zhang X., Ma P., Yang B., Xu S., Lang J. (2023). High-voltage electrochemical double layer capacitors enabled by polymeric ionic liquid. Electrochim. Acta.

[61] Eftekhari A. (2017). Supercapacitors utilising ionic liquids. Energy Storage Mater..

[62] Feng J., Wang Y., Xu Y., Sun Y., Tang Y., Yan X. (2021). Ion regulation of ionic liquid electrolytes for supercapacitors. Energy Environ. Sci..

[63] Nakamoto H., Noda A., Hayamizu K., Hayashi S., Hamaguchi H.-o., Watanabe M. (2007). Proton-Conducting Properties of a Brønsted Acid—Base Ionic Liquid and Ionic Melts Consisting of Bis(trifluoromethanesulfonyl)imide and Benzimidazole for Fuel Cell Electrolytes. J. Phys. Chem. C.

[64] Elwan H.A., Thimmappa R., Mamlouk M., Scott K. (2021). Applications of poly ionic liquids in proton exchange membrane fuel cells: A review. J. Power Sources.

[65] Hou H., Schütz H.M., Giffin J., Wippermann K., Gao X., Mariani A., Passerini S., Korte C. (2021). Acidic Ionic Liquids Enabling Intermediate Temperature Operation Fuel Cells. ACS Appl. Mater. Interfaces.

[66] Nancarrow P., Al-Othman A., Mital D.K., Döpking S. (2021). Comprehensive analysis and correlation of ionic liquid conductivity data for energy applications. Energy.

[67] Lu W., Fadeev A.G., Qi B., Mattes B.R. (2004). Fabricating Conducting Polymer Electrochromic Devices Using Ionic Liquids. J. Electrochem. Soc..

[68] Kim H., Kim K., Choi D., Lee C.S. (2017). Evaluation of a reliable electrochromic device based on PEDOT:PSS-TiO 2 heterostructure fabricated at low temperature. Ionics.

[69] Rochefort D. (2019). Enabling new electrochemical methods with redox-active ionic liquids. Curr. Opin. Electrochem..

[70] Sun L., Zhuo K., Chen Y., Du Q., Zhang S., Wang J. (2022). Ionic Liquid-Based Redox Active Electrolytes for Supercapacitors. Adv. Funct. Mater..

[71] Tahara H., Baba R., Iwanaga K., Sagara T., Murakami H. (2017). Electrochromism of a Bipolar Reversible Redox-Active Ferrocene-Viologen Linked Ionic Liquid. Chem. Commun..

[72] Zhao J., Chen Q., Wang Z., Zheng J., Xu C. (2024). All-ionic liquid electrochromic devices based on viologen-type and ferrocene-type room-temperature ionic liquids with temperature adaptability and environmental friendliness. Sol. Energy Mater. Sol. Cells.

[73] Branco A., Branco L.C., Pina F. (2011). Electrochromic and magnetic ionic liquids. Chem. Commun..

[74] Branco A., Belchior J., Branco L.C., Pina F. (2013). Intrinsically electrochromic ionic liquids based on vanadium oxides: Illustrating liquid electrochromic cells. RSC Adv..

[75] Jordão N., Cruz H., Branco A., Pina F., Branco L.C. (2015). Electrochromic Devices Based on Disubstituted Oxo-Bipyridinium Ionic Liquids. ChemPlusChem.

[76] Hwang E., Seo S., Bak S., Lee H., Min M., Lee H. (2014). An Electrolyte-Free Flexible Electrochromic Device Using Electrostatically Strong Graphene Quantum Dot–Viologen Nanocomposites. Adv. Mater..

[77] Lu H.-C., Kao S.-Y., Chang T.-H., Kung C.-W., Ho K.-C. (2016). An electrochromic device based on Prussian blue, self-immobilized vinyl benzyl viologen, and ferrocene. Sol. Energy Mater. Sol. Cells.

[78] Gélinas B., Das D., Rochefort D. (2017). Air-Stable, Self-Bleaching Electrochromic Device Based on Viologen- and Ferrocene-Containing Triflimide Redox Ionic Liquids. ACS Appl. Mater. Interfaces.

[79] Tahara H., Uranaka K., Hirano M., Ikeda T., Sagara T., Murakami H. (2018). Electrochromism of Ferrocene- and Viologen-Based Redox-Active Ionic Liquids Composite. ACS Appl. Mater. Interfaces.

[80] Song R., Li G., Zhang Y., Rao B., Xiong S., He G. (2021). Novel electrochromic materials based on chalcogenoviologens for smart windows, E-price tag and flexible display with improved reversibility and stability. Chem. Eng. J..

[81] Fan M.-S., Lee C.-P., Vittal R., Ho K.-C. (2017). A novel ionic liquid with stable radical as the electrolyte for hybrid type electrochromic devices. Sol. Energy Mater. Sol. Cells.

[82] Kim J., Kim D., Jang H., Auh Y., Kim B., Kim E. (2022). Transparent photo-electrochromic capacitive windows with a Bi-dopant redox ionic liquids. Chem. Eng. J..

[83] Bai Z., Wu X., Fang R., Lu Z., Hou C., Zhang Q., Li Y., Li K., Wang H. (2024). Divalent Viologen Cation-Based Ionogels Facilitate Reversible Intercalation of Anions in PProDOT-Me_2_ for Flexible Electrochromic Displays. Adv. Funct. Mater..

[84] Wu Y., Li Y., Wang Y., Liu Q., Chen Q., Chen M. (2022). Advances and prospects of PVDF based polymer electrolytes. J. Energy Chem..

[85] Gao K., Ju S., Li S., Zhang S., Liu J., Yang T., Lv J., Yu W., Zhang Z. (2023). Decoupling Electrochromism and Energy Storage for Flexible Quasi-Solid-State Aqueous Electrochromic Batteries with High Energy Density. ACS Nano.

[86] Xu F., Li H., Li Y. (2024). Sea Cucumber-Inspired Polyurethane Demonstrating Record-Breaking Mechanical Properties in Room-Temperature Self-Healing Ionogels. Adv. Mater..

[87] Leones R., Sabadini R.C., Sentanin F.C., Esperança J.M.S.S., Pawlicka A., Silva M.M. (2017). Polymer electrolytes for electrochromic devices through solvent casting and sol-gel routes. Sol. Energy Mater. Sol. Cells.

[88] Danine A., Manceriu L., Fargues A., Rougier A. (2017). Eco-friendly redox mediator gelatin-electrolyte for simplified TiO2-viologen based electrochromic devices. Electrochim. Acta.

[89] Alves R., Fidalgo-Marijuan A., Campos-Arias L., Gonçalves R., Silva M.M., Del Campo F.J., Costa C.M., Lanceros-Mendez S. (2022). Solid Polymer Electrolytes Based on Gellan Gum and Ionic Liquid for Sustainable Electrochromic Devices. ACS Appl. Mater. Interfaces.

[90] Alves R., Fidalgo-Marijuan A., Salado M., Gonçalves R., Silva M.M., Bazán B., del Campo F.J., Costa C.M., Lanceros-Mendez S. (2023). Agar-Based Solid Polymer Electrolyte-Containing Ionic Liquid for Sustainable Electrochromic Devices. ACS Sustain. Chem. Eng..

[91] Serra J.P., Salado M., Correia D.M., Gonçalves R., Del Campo F.J., Lanceros-Mendez S., Costa C.M. (2023). High-Performance Sustainable Electrochromic Devices Based on Carrageenan Solid Polymer Electrolytes with Ionic Liquid. ACS Appl. Eng. Mater..

[92] Zhang H., Shi W., Cheng H., Chen S., Wang L. (2018). Effect of ionic liquid on crystallization kinetics and crystal form transition of poly(vinylidene fluoride) blends. J. Therm. Anal. Calorim..

[93] Marcilla R., Alcaide F., Sardon H., Pomposo J.A., Pozo-Gonzalo C., Mecerreyes D. (2006). Tailor-made polymer electrolytes based upon ionic liquids and their application in all-plastic electrochromic devices. Electrochem. Commun..

[94] Pozo-Gonzalo C., Mecerreyes D., Pomposo J.A., Salsamendi M., Marcilla R., Grande H., Vergaz R., Barrios D., Sánchez-Pena J.M. (2008). All-plastic electrochromic devices based on PEDOT as switchable optical attenuator in the near IR. Sol. Energy Mater. Sol. Cells.

[95] Poh W.C., Eh A.L.-S., Wu W., Guo X., Lee P.S. (2022). Rapidly Photocurable Solid-State Poly(ionic liquid) Ionogels For Thermally Robust and Flexible Electrochromic Devices. Adv. Mater..

[96] Chen F., Ren Y., Guo J., Yan F. (2017). Thermo- and electro-dual responsive poly(ionic liquid) electrolyte based smart windows. Chem. Commun..

[97] Rathod P.V., Puguan J.M.C., Kim H. (2021). Self-bleaching dual responsive poly(ionic liquid) with optical bistability toward climate-adaptable solar modulation. Chem. Eng. J..

[98] Sun F., Sik Kim K., Yeon Eom S., Won Choi J., Kim E.J., Abdul Raheem A., Jeon S.-P., Gi Seong D., Ahn S.-K., Kyu Park S. (2024). Stretchable interconnected modular electrochromic devices enabled by self-healing, self-adhesive, and ion-conducting polymer electrolyte. Chem. Eng. J..

[99] Su X., Xu X.-P., Ji Z.-Q., Wu J., Ma F., Fan L.-Z. (2024). Polyethylene Oxide-Based Composite Solid Electrolytes for Lithium Batteries: Current Progress, Low-Temperature and High-Voltage Limitations, and Prospects. Electrochem. Energy Rev..

[100] Ding P., Lin Z., Guo X., Wu L., Wang Y., Guo H., Li L., Yu H. (2021). Polymer electrolytes and interfaces in solid-state lithium metal batteries. Mater. Today.

[101] Brazier A., Appetecchi G.B., Passerini S., Surca Vuk A., Orel B., Donsanti F., Decker F. (2007). Ionic liquids in electrochromic devices. Electrochim. Acta.

[102] Bircan H., Seshadri V., Padilla J., Invernale M., Otero T.F., Sotzing G.A. (2008). Use of polymer/ionic liquid plasticizers as gel electrolytes in electrochromic devices. J. Phys. Conf. Ser..

[103] Desai S., Shepherd R.L., Innis P.C., Murphy P., Hall C., Fabretto R., Wallace G.G. (2010). Gel electrolytes with ionic liquid plasticiser for electrochromic devices. Electrochim. Acta.

[104] Zanarini S., Garino N., Nair J.R., Francia C., Wojcik P.J., Pereira L., Fortunato E., Martins R., Bodoardo S., Penazzi N. (2014). Contrast Enhancement in Polymeric Electrochromic Devices Encompassing Room Temperature Ionic Liquids. Int. J. Electrochem. Sci..

[105] Kim J.W., Myoung J.M. (2019). Flexible and Transparent Electrochromic Displays with Simultaneously Implementable Subpixelated Ion Gel-Based Viologens by Multiple Patterning. Adv. Funct. Mater..

[106] Chaudhary A., Poddar M., Pathak D.K., Misra R., Kumar R. (2020). Electron Donor Ferrocenyl Phenothiazine: Counter Ion for Improving All-Organic Electrochromism. ACS Appl. Electron. Mater..

[107] Pathak D.K., Chaudhary A., Tanwar M., Goutam U.K., Kumar R. (2020). Nano-cobalt oxide/viologen hybrid solid state device: Electrochromism beyond chemical cell. Appl. Phys. Lett..

[108] Fang R., Bai Z., Wu X., Fan Q., Bao B., Hou C., Zhang Q., Li Y., Li K., Wang H. (2024). Electro-Induced Self-Reduction TiO _2_ in Viologen-Based Ionogels for Multi-Color Electrochromic Displays. Adv. Opt. Mater..

[109] Gong H., Li A., Fu G., Zhang M., Zheng Z., Zhang Q., Zhou K., Liu J., Wang H. (2023). Ultrathin flexible electrochromic devices enabled by highly transparent ion-conducting films. J. Mater. Chem. A.

[110] Yang G., Li X., He Y., Ma J., Ni G., Zhou S. (2018). From nano to micro to macro: Electrospun hierarchically structured polymeric fibers for biomedical applications. Prog. Polym. Sci..

[111] Jia P., Yee W.A., Xu J., Toh C.L., Ma J., Lu X. (2011). Thermal stability of ionic liquid-loaded electrospun poly(vinylidene fluoride) membranes and its influences on performance of electrochromic devices. J. Membr. Sci..

[112] Zhou R., Liu W., Kong J., Zhou D., Ding G., Leong Y.W., Pallathadka P.K., Lu X. (2014). Chemically cross-linked ultrathin electrospun poly(vinylidene fluoride-co-hexafluoropropylene) nanofibrous mats as ionic liquid host in electrochromic devices. Polymer.

[113] Zhou R., Pramoda K.P., Liu W., Zhou D., Ding G., He C., Leong Y.W., Lu X. (2014). Electrospun poly(vinylidene fluoride) copolymer/octahydroxy-polyhedral oligomeric silsesquioxane nanofibrous mats as ionic liquid host: Enhanced salt dissociation and its function in electrochromic device. Electrochim. Acta.

[114] Zhou R., Liu W., Yao X., Leong Y.W., Lu X. (2015). Poly(vinylidene fluoride) nanofibrous mats with covalently attached SiO_2_ nanoparticles as an ionic liquid host: Enhanced ion transport for electrochromic devices and lithium-ion batteries†. J. Mater. Chem. A.

[115] Zhou R., Liu W., Leong Y.W., Xu J., Lu X. (2015). Sulfonic Acid- and Lithium Sulfonate-Grafted Poly(Vinylidene Fluoride) Electrospun Mats As Ionic Liquid Host for Electrochromic Device and Lithium-Ion Battery. ACS Appl. Mater. Interfaces.

[116] Moon H.C., Kim C.-H., Lodge T.P., Frisbie C.D. (2016). Multicolored, Low-Power, Flexible Electrochromic Devices Based on Ion Gels. ACS Appl. Mater. Interfaces.

[117] Oh H., Seo D.G., Yun T.Y., Kim C.Y., Moon H.C. (2017). Voltage-Tunable Multicolor, Sub-1.5 V, Flexible Electrochromic Devices Based on Ion Gels. ACS Appl. Mater. Interfaces.

[118] Xing G., Kuang G., Tao Y., Wang Y., Kang Y., Guo Y., Zhang S. (2022). Ultra-strong ionic liquid-based polymer composite electrolyte for high performance electrochromic devices. Sol. Energy Mater. Sol. Cells.

[119] Kavanagh A., Copperwhite R., Oubaha M., Owens J., McDonagh C., Diamond D., Byrne R. (2011). Photo-patternable hybrid ionogels for electrochromic applications. J. Mater. Chem..

[120] Kavanagh A., Fraser K.J., Byrne R., Diamond D. (2012). An Electrochromic Ionic Liquid: Design, Characterization, and Performance in a Solid-State Platform. ACS Appl. Mater. Interfaces.

[121] Kim Y.M., Lee W.Y., Choi W.Y., Moon H.C. (2020). Impact of Chain Flexibility of Copolymer Gelators on Performance of Ion Gel Electrolytes for Functional Electrochemical Devices. J. Ind. Eng. Chem..

[122] Pande G.K., Choi J.H., Lee J.-E., Kim Y.E., Choi J.H., Choi H.W., Chae H.G., Park J.S. (2020). Octa-Viologen Substituted Polyhedral Oligomeric Silsesquioxane Exhibiting Outstanding Electrochromic Performances. Chem. Eng. J..

[123] Chen W., Liu S., Guo L., Zhang G., Zhang H., Cao M., Wu L., Xiang T., Peng Y. (2021). A Self-Healing Ionic Liquid-Based Ionically Cross-Linked Gel Polymer Electrolyte for Electrochromic Devices. Polymers.

[124] Jang Y.J., Kim S.Y., Kim Y.M., Lee J.K., Moon H.C. (2021). Unveiling the diffusion-controlled operation mechanism of all-in-one type electrochromic supercapacitors: Overcoming slow dynamic response with ternary gel electrolytes. Energy Storage Mater..

[125] Xing G., Wu L., Kuang G., Ma T., Chen Z., Tao Y., Kang Y., Zhang S. (2022). Integration of high surface-energy electrochromic polymer with in-situ polymerized quasi-solid electrolyte for efficient electrochromism. Electrochim. Acta.

[126] Zhang Y., Guo M., Li G., Chen X., Liu Z., Shao J., Huang Y., He G. (2022). Ultrastable Viologen Ionic Liquids-Based Ionogels for Visible Strain Sensor Integrated with Electrochromism, Electrofluorochromism, and Strain Sensing. CCS Chem..

[127] Zhou X., Zhou K., Tang L., Chen Z., Hu Q., Gao J., Zhang Y., Zhang J., Zhang S. (2024). A Strong and Highly Transparent Ionogel Electrolyte Enabled by In Situ Polymerization-Induced Microphase Separation for High-Performance Electrochromic Devices. Macromol. Rapid Commun..

[128] Sydam R., Deepa M., Srivastava A.K. (2012). Electrochromic device response controlled by an in situ polymerized ionic liquid based gel electrolyte. RSC Adv..

[129] He X., Cheng H., Yue S., Ouyang J. (2020). Quasi-solid state nanoparticle/(ionic liquid) gels with significantly high ionic thermoelectric properties. J. Mater. Chem. A.

[130] Zhang F., Dong G., Liu J., Ye S., Diao X. (2017). Polyvinyl butyral-based gel polymer electrolyte films for solid-state laminated electrochromic devices. Ionics.

[131] Okutan M., Evecan D., Yıldırım S., Özkan Zayim E., Deligöz H. (2020). Investigating the effect of electrolyte types with various ionic liquids on the electrochromic performance of PEDOT:PSS based LbL multilayers. Microelectron. Eng..

[132] Koo J., Amoli V., Kim S.Y., Lee C., Kim J., Park S.-M., Kim J., Ahn J.M., Jung K.J., Kim D.H. (2020). Low-power, deformable, dynamic multicolor electrochromic skin. Nano Energy.

[133] Oh S.-J., Bae J.W. (2023). All-in-One plasticized Ionogel-based stretchable electrochromic devices. Chem. Eng. J..

[134] Li J., Liu W., Wei Y., Yan Y. (2023). SiO2: A Novel Electrolyte for High-Performance All-Solid-State Electrochromic Devices. ACS Sustain. Chem. Eng..

[135] Wang H., Wang J., Shi Q., Su Y., Tang P., Huang S., Lin S., Dai M. (2023). Influence of LiPON thickness on the electro-optical performance of inorganic all-solid-state electrochromic devices. Sol. Energy Mater. Sol. Cells.

[136] Song Y., Cheng B., Cheng H., Meng Z., Liu D. (2024). All-Solid-State Infrared Electrochromic Devices Based on Thin Metal Films. ACS Appl. Mater. Interfaces.

[137] Jia Hanxiang S.Z.H.A.J.I.N.P.C.A.O.X. (2021). Sandwich Structured Electrolyte of High Sputtering Efficiency for All-solid-state Electrochromic Devices by Optical Design. J. Inorg. Mater..

[138] Lin C.-C., Chen P.-H., Chen M.-C., Wang M.-C., Yang C.-C., Huang H.-C., Wu C.-W., Chou S.-Y., Tsai T.-M., Chang T.-C. (2022). Improved diffusion and storage of lithium ions via recrystallization induced conducting pathways in a li:Ta2O5-based electrolyte for all-solid-state electrochromic devices with enhanced performance. Nanotechnology.

[139] Chen H.-C., Chen Y.-R., Liu T.-F. (2021). Photoelectrochemical performance of a UV-cured all-solid-state complementary ITO/WO_3_/Ta_2_O_5_/electrolyte/NiO/ITO electrochromic device deposited by ion-beam assisted electron-beam evaporation. Electrochim. Acta.

[140] Zhang T., Mu X., Li Y., Cong S., Zheng S., Huang R., Geng F., Zhao Z. (2024). Optical-Cavity-Incorporated Colorful All-Solid-State Electrochromic Devices for Dual Anti-Counterfeiting. Adv. Mater..

[141] Zhang J., Tu J.P., Xia X.H., Qiao Y., Lu Y. (2009). An all-solid-state electrochromic device based on NiO/WO_3_ complementary structure and solid hybrid polyelectrolyte. Sol. Energy Mater. Sol. Cells.

[142] Li N., Bao S., Zhang Q., Xie L., Jin P. (2018). Wide-Band Reflection-Type, All-Solid-State Switchable Mirror Composed of WO_3_–Mg_4_Ni Thin Films and Proton-Conductive Polymer Electrolytes. ChemistrySelect.

[143] Rong D., Wu Y., Wang W., Shang X., Wang S., Wang S. (2024). Polyvinyl Butyral Solid Electrolyte Film and Its Electrochromic Laminated Safety Glass. ACS Appl. Mater. Interfaces.

[144] Lin C.-L., Chen A. (2022). Electrochromic devices composed of polyaniline and tungsten trioxide thin films with succinonitrile/polyethylene glycol solid-state composite electrolyte. Surf. Coat. Technol..

[145] Liu H., Wang M., Wang X., Pawlicka A., Diao X. (2024). In situ synthesis and structural morphology analysis of 3D porous hierarchical V_2_O_5_ films for transmissive-to-black all-solid-state electrochromic devices. Chem. Eng. J..

[146] Vijayaraghavan S., Raj N., Kumar M.M., Mohan P.A., Ak N.K. (2024). Ionic conductivity and dielectric characteristics of a Li incorporated PVA electrolyte membrane and a study of a fully solid-state electrochromic device based on it. Electrochim. Acta.

[147] Ganesh G.P.T., Deb B. (2017). Designing an All-Solid-State Tungsten Oxide Based Electrochromic Switch with a Superior Cycling Efficiency. Adv. Mater. Interfaces.

[148] Wu X., Wang Q., Zhang W., Wang Y., Chen W. (2016). Preparation of all-solid-state supercapacitor integrated with energy level indicating functionality. Synth. Met..

[149] Du Q., Fu X., Liu S., Niu L. (2012). The effect of ionic liquid fragment on the performance of polymer electrolytes. Polym. Int..

[150] Aubert P.-H., Argun A.A., Cirpan A., Tanner D.B., Reynolds J.R. (2004). Microporous Patterned Electrodes for Color-Matched Electrochromic Polymer Displays. Chem. Mater..

[151] Rauh R.D., Wang F., Reynolds J.R., Meeker D.L. (2001). High coloration efficiency electrochromics and their application to multi-color devices. Electrochim. Acta.

[152] Mecerreyes D., Marcilla R., Ochoteco E., Grande H., Pomposo J.A., Vergaz R., Sánchez Pena J.M. (2004). A simplified all-polymer flexible electrochromic device. Electrochim. Acta.

[153] Tran-Van F., Beouch L., Vidal F., Yammine P., Teyssié D., Chevrot C. (2008). Self-supported semi-interpenetrating polymer networks for new design of electrochromic devices. Electrochim. Acta.

[154] De Paoli M.A., Casalbore-Miceli G., Girotto E.M., Gazotti W.A. (1999). All polymeric solid state electrochromic devices. Electrochim. Acta.

[155] Wang J., Zhao Y., Zhuo K., Lin R. (2003). Viscosity Properties of Electrolytes in Propylene Carbonate Based Lithium Battery Electrolyte Solutions. Z. Phys. Chem..

[156] Yu G., Zhao D., Wen L., Yang S., Chen X. (2012). Viscosity of ionic liquids: Database, observation, and quantitative structure-property relationship analysis. AlChE J..

[157] Eftekhari A., Liu Y., Chen P. (2016). Different roles of ionic liquids in lithium batteries. J. Power Sources.

[158] Simonis E.D., Blanchard G.J. (2024). Evaluating the contributions to conductivity in room temperature ionic liquids. Phys. Chem. Chem. Phys..

[159] Wu K.-J., Luo H., Yang L. (2016). Structure-based model for prediction of electrical conductivity of pure ionic liquids. AlChE J..

